# Supplementary material for: Correlation between pose estimation features regarding movements towards the midline in early infancy
Source: PLoS One. 2024 Feb 28;19(2):e0299758. doi: 10.1371/journal.pone.0299758 (PMC10901309; doi:10.1371/journal.pone.0299758)
Supplement: S1 Table — This table included gestational age (GA) and anthropometrics at birth. Percentiles are presented according to the 2006 WHO Child Growth Standards: 0–6 months (weight and length) and 0–13 weeks (head circumference) [32]. The Apgar scores at 1 and 5 minutes, along with the age at the time of video collection, are provided for each infant. (DOCX) [file pone.0299758.s001.docx]

**S1 Table Infant demographic data (N = 20)** This table included gestational age (GA) and anthropometrics at birth. Percentiles are presented according to the 2006 WHO Child Growth Standards: 0-6 months (weight and length) and 0-13 weeks (head circumference) [32]. The Apgar scores at 1 and 5 minutes, along with the age at the time of video collection, are provided for each infant.

| **Subject** | **Gender** | **Gestational age (weeks)** | **Birth Weight** | | **Birth Length** | | **Birth Head**  **Circumference** | | **Apgar score**  **(min 1, 5)** | **Video collection age**  (Estimated weeks on top row and days PTA were reported in table) | | | | | | | | |
| --- | --- | --- | --- | --- | --- | --- | --- | --- | --- | --- | --- | --- | --- | --- | --- | --- | --- | --- |
|  |  |  | g | Percentile | cm | Percentile | cm | Percentile |  | 8 | 9 | 10 | 11 | 12 | 13 | 14 | 15 | 16 |
| 1 | G | 39 | 2720 | P3-P15 | 47 | P3-P15 | 34 | P50-P85 | (8,9) |  |  | 73 | 80 | 87 | 94 |  | 102 | 113 |
| 2 | G | 38 | 2850 | P15-P50 | 49 | P15-P50 | 33 | P15-P50 | (7,9) |  | 63 | 69 | 77 |  |  |  |  |  |
| 3 | G | 38 4/7 | 3270 | P50-P85 | 49 | P15-P50 | 34 | P50-P85 | (10,10) |  |  | 70 | 78 | 86 | 94 |  | 102 | 112 |
| 4 | G | 37 | 2550 | P3-P15 | 48 | P15-P50 | 31 | <P3 | (10,10) |  | 62 |  |  |  |  |  |  |  |
| 5 | B | 38 1/7 | 3610 | P50-P85 | 50 | P50 | 35.5 | P50-P85 | (9,9) | 56 | 63 | 68 | 77 |  |  |  |  |  |
| 6 | B | 37 | 3060 | P15-P50 | 50 | P50 | 35 | P50-P85 | (8,10) |  | 62 |  | 78 |  | 93 |  |  |  |
| 7 | G | 39 4/7 | 3390 | P50-P85 | 51 | P85 | 34.5 | P50-P85 | (9,10) | 53 |  |  |  |  | 90 | 95 |  |  |
| 8 | G | 40 1/7 | 3900 | P85-P97 | N/A | | | | |  |  |  | 76 | 83 | 90 | 99 | 105 | 113 |
| 9 | B | 38 2/7 | 2740 | P3-P15 | 48 | P15 | 34 | P15-P50 | (9,10) | 59 | 65 | 72 | 79 |  |  | 95 | 103 |  |
| 10 | B | 38 2/7 | 3270 | P15-P50 | 47 | P3-P15 | 36.5 | P85-P97 | (9,10) | 53 | 63 | 76 |  | 94 |  |  |  |  |
| 11 | B | 38 | 3180 | P15-P50 | 50 | P50 | 33.5 | P15-P50 | (8,9) |  |  |  |  |  |  | 100 |  |  |
| 12 | B | 39 6/7 | 3080 | P15-P50 | 49 | P15-P50 | 34.5 | P50 | (9,10) |  | 61 | 71 | 77 | 85 | 94 |  | 104 |  |
| 13 | B | 39 | 2790 | P3-P15 | 49 | P15-P50 | 33 | P3-P15 | (10,10) |  | 63 | 70 | 77 | 84 | 92 | 98 | 105 |  |
| 14 | B | 39 1/7 | 3205 | P15-P50 | 55 | >P97 | 32.5 | P3-P15 | (9,9) |  |  |  | 76 | 85 | 92 | 99 |  |  |
| 15 | B | 38 3/7 | 2686 | P3-P15 | 47 | P3-P15 | 32.5 | P3-P15 | (9,10) |  |  |  |  |  | 88 |  |  |  |
| 16 | G | 38 4/7 | 2710 | P3-P15 | 49 | P15-P50 | 34 | P50-P85 | (9,10) | 53 | 60 | 67 | 75 | 82 | 89 | 95 | 103 |  |
| 17 | G | 40 1/7 | 3150 | P15-P50 | 50 | P50-P85 | 35 | P50-P85 | (8,9) | 57 |  | 71 | 78 | 84 | 91 | 98 | 105 | 112 |
| 18 | G | 39 | 3310 | P50-P85 | 50 | P50-P85 | 34 | P50-P85 | (7,9) | 56 | 63 | 71 | 79 | 85 | 91 | 98 | 106 |  |
| 19 | B | 39 5/7 | 3255 | P15-P50 | 50 | P50 | 35.5 | P50-P85 | (9,10) | 54 |  |  |  |  |  | 96 |  |  |
| 20 | G | 38 1/7 | 2830 | P15-P50 | 48 | P15-P50 | 33 | P15-P50 | (8,10) | 57 | 65 | 71 | 78 | 85 | 92 | 99 |  |  |

G = Girl, B = Boy, g = grams, cm = centimetres, min = minute, PTA = post-term age, P3 = 3rd percentile, P15 = 15th percentile, P50 = 50th percentile, P85 = 85th percentile, P97 = 97th percentile
