# Supplementary material for: Correlation between pose estimation features regarding movements towards the midline in early infancy
Source: PLoS One. 2024 Feb 28;19(2):e0299758. doi: 10.1371/journal.pone.0299758 (PMC10901309; doi:10.1371/journal.pone.0299758)
Supplement: Attachment — Submitted filename: PONE-D-23-29999 Annotated.pdf [file pone.0299758.s003.pdf]

PLOS ONE

Correlation between pose estimation features regarding movements towards the midline in early infancy

--Manuscript Draft--

**Manuscript Number:**	PONE-D-23-29999
**Article Type:**	Research Article
**Full Title:**	Correlation between pose estimation features regarding movements towards the midline in early infancy
**Short Title:**	Early infancy movement toward midline and pose estimation
**Corresponding Author:**	Hirotaka Gima, Ph.D.<br>Tokyo Metropolitan University Graduate School of Health Sciences Faculty of Health Sciences: Tokyo Toritsu Daigaku Kenko Fukushi Gakubu Daigakuin Ningen Kenko Kagaku Kenkyuka<br>Arakawa, Tokyo JAPAN
**Keywords:**	movement toward midline; 2D pose estimation; paediatric physical therapy; movement assessment; infants
**Abstract:**	In infants, spontaneous movement towards the midline (MTM) indicates the initiation of anti-gravity ability development. Markerless 2D pose estimation is a cost-effective, time-efficient, and quantifiable alternative to movement assessment. We aimed to establish correlations between pose estimation features and MTM in early-age infants. Ninety-four infant videos were analysed to calculate the percentage and rate of MTM occurrence. Pose estimation processed the videos and determined the distances and areas using the wrist and ankle landmark coordinates. We collected data using video recordings from 20 infants aged 8–16 weeks PTA. Correlations between MTM observations and distance values were evaluated. Differences in areas between groups of videos showing MTM and no MTM in the total, lower-limb, and upper-limb categories were examined. MTM observations revealed common occurrences of hand-to-trunk and foot-to-foot movements. Weak correlations were noted between limb distances to the midbody imaginary line and the MTM occurrence values. Lower MTM showed significant differences in the lower part (p=0.003) and whole area (p=0.001). Video recording by guardians could extract features using 2D pose estimation, assisting in the early identification of MTM in infants. Further research is required to assess the diversity of MTM motor behaviour, and later developmental skills, and collect data from at-risk infants.
**Order of Authors:**	NISASRI SERMPON
	Hirotaka Gima
**Additional Information:**	
**Question**	**Response**
**Financial Disclosure**<br><br>Enter a financial disclosure statement that describes the sources of funding for the work included in this submission. Review the submission guidelines for detailed requirements. View published research articles from *PLOS ONE* for specific examples.<br><br>This statement is required for submission and **will appear in the published article** if the submission is accepted. Please make sure it is accurate.	This research was funded by the Tokyo Human Resource Fund for the City Diplomacy Scholar and the MEXT Grant-in-Aid for Scientific Research (no.21K11502 to H.G.). The funders had no role in study design, data collection and analysis, decision to publish, or preparation of the manuscript.

Powered by Editorial Manager® and ProduXion Manager® from Aries Systems Corporation

**Unfunded studies**<br>Enter: *The author(s) received no specific funding for this work.*<br><br>**Funded studies**<br>Enter a statement with the following details:<br>• Initials of the authors who received each award<br>• Grant numbers awarded to each author<br>• The full name of each funder<br>• URL of each funder website<br>• Did the sponsors or funders play any role in the study design, data collection and analysis, decision to publish, or preparation of the manuscript?<br>• **NO** - Include this sentence at the end of your statement: *The funders had no role in study design, data collection and analysis, decision to publish, or preparation of the manuscript.*<br>• **YES** - Specify the role(s) played.<br><br>* typeset	
**Competing Interests**<br><br>Use the instructions below to enter a competing interest statement for this submission. On behalf of all authors, disclose any competing interests that could be perceived to bias this work—acknowledging all financial support and any other relevant financial or non-financial competing interests.<br><br>This statement is required for submission and **will appear in the published article** if the submission is accepted. Please make sure it is accurate and that any funding sources listed in your Funding Information later in the submission form are also declared in your Financial Disclosure statement.<br><br>View published research articles from *PLOS ONE* for specific examples.	The authors have declared that no competing interests exist.

Powered by Editorial Manager® and ProduXion Manager® from Aries Systems Corporation

**NO authors have competing interests**<br><br>Enter: *The authors have declared that no competing interests exist.*<br><br>**Authors with competing interests**<br><br>Enter competing interest details beginning with this statement:<br><br>*I have read the journal's policy and the authors of this manuscript have the following competing interests: [insert competing interests here]*<br><br>* typeset	
**Ethics Statement**<br><br>Enter an ethics statement for this submission. This statement is required if the study involved:<br><br>• Human participants<br>• Human specimens or tissue<br>• Vertebrate animals or cephalopods<br>• Vertebrate embryos or tissues<br>• Field research<br><br>Write "N/A" if the submission does not require an ethics statement.<br><br>General guidance is provided below. Consult the submission guidelines for detailed instructions. **Make sure that all information entered here is included in the Methods section of the manuscript.**	The study was conducted in accordance with the Declaration of Helsinki and approved by the Ethics Committee of Graduated School of Human Health Science, Tokyo Metropolitan University (no. 21092). Authorisation was obtained and informed consent was acquired from the participants' parents or legal guardians.

**Format for specific study types**<br><br>**Human Subject Research (involving human participants and/or tissue)**<br>• Give the name of the institutional review board or ethics committee that approved the study<br>• Include the approval number and/or a statement indicating approval of this research<br>• Indicate the form of consent obtained (written/oral) or the reason that consent was not obtained (e.g. the data were analyzed anonymously)<br><br>**Animal Research (involving vertebrate animals, embryos or tissues)**<br>• Provide the name of the Institutional Animal Care and Use Committee (IACUC) or other relevant ethics board that reviewed the study protocol, and indicate whether they approved this research or granted a formal waiver of ethical approval<br>• Include an approval number if one was obtained<br>• If the study involved *non-human primates*, add *additional details* about animal welfare and steps taken to ameliorate suffering<br>• If anesthesia, euthanasia, or any kind of animal sacrifice is part of the study, include briefly which substances and/or methods were applied<br><br>**Field Research**<br><br>Include the following details if this study involves the collection of plant, animal, or other materials from a natural setting:<br>• Field permit number<br>• Name of the institution or relevant body that granted permission	
**Data Availability**<br><br>Authors are required to make all data underlying the findings described fully available, without restriction, and from the time of publication. PLOS allows rare exceptions to address legal and ethical concerns. See the PLOS Data Policy and FAQ for detailed information.	Yes - all data are fully available without restriction

A Data Availability Statement describing where the data can be found is required at submission. Your answers to this question constitute the Data Availability Statement and **will be published in the article**, if accepted.<br><br>**Important:** Stating 'data available on request from the author' is not sufficient. If your data are only available upon request, select 'No' for the first question and explain your exceptional situation in the text box.<br><br>Do the authors confirm that all data underlying the findings described in their manuscript are fully available without restriction?	
**Describe where the data may be found in full sentences. If you are copying our sample text, replace any instances of XXX with the appropriate details.**<br><br>• If the data are **held or will be held in a public repository**, include URLs, accession numbers or DOIs. If this information will only be available after acceptance, indicate this by ticking the box below. For example: *All XXX files are available from the XXX database (accession number(s) XXX, XXX.*).<br>• If the data are all contained **within the manuscript and/or Supporting Information files**, enter the following: *All relevant data are within the manuscript and its Supporting Information files.*<br>• If neither of these applies but you are able to provide **details of access elsewhere**, with or without limitations, please do so. For example:<br><br>*Data cannot be shared publicly because of [XXX]. Data are available from the XXX Institutional Data Access / Ethics Committee (contact via XXX) for researchers who meet the criteria for access to confidential data.*<br><br>*The data underlying the results presented in the study are available from (include the name of the third party*	All data files are available from the figshare database (DOI: 10.6084/m9.figshare.24143679). All relevant data are within the manuscript and Supporting Information files.

and contact information or URL).<br>• This text is appropriate if the data are owned by a third party and authors do not have permission to share the data.<br><br>* typeset	
Additional data availability information:	Tick here if the URLs/accession numbers/DOIs will be available only after acceptance of the manuscript for publication so that we can ensure their inclusion before publication.

Powered by Editorial Manager® and ProduXion Manager® from Aries Systems Corporation

Manuscript

Click here to access/download;Manuscript;002_Manuscript_20231005.docx

Correlation between pose estimation features regarding movements towards the midline in early infancy

Nisasri Sermpon [1,2], Hirotaka Gima[1]*

[1]Department of Physical Therapy, Graduate School of Human Health Sciences, Tokyo Metropolitan University, Arakawa, Tokyo, Japan

[2]Faculty of Physical Therapy, Mahidol University, Salaya, Nakhon Pathom, Thailand

*Corresponding author

Email: gima@tmu.ac.jp (HG)

Abstract

In infants, spontaneous movement towards the midline (MTM) indicates the initiation of anti-gravity ability development. Markerless 2D pose estimation is a cost-effective, time-efficient, and quantifiable alternative to movement assessment. We aimed to establish correlations between pose estimation features and MTM in early-age infants. Ninety-four infant videos were analysed to calculate the percentage and rate of MTM occurrence. Pose estimation processed the videos and determined the distances and areas using the wrist and ankle landmark coordinates. We collected data using video recordings from 20 infants aged 8–16 weeks PTA. Correlations between MTM observations and distance values were evaluated. Differences in areas between groups of videos showing MTM and no MTM in the total, lower-limb, and upper-limb categories were examined. MTM observations revealed common occurrences of hand-to-trunk and foot-to-foot movements. Weak correlations were noted between limb distances to the midbody imaginary line and the MTM occurrence values. Lower MTM showed significant differences in the lower part (p=0.003) and whole area (p=0.001). Video recording by guardians could extract features using 2D pose estimation, assisting in the early identification of MTM in infants. Further research is required to assess the diversity of MTM motor behaviour, and later developmental skills, and collect data from at-risk infants.

Introduction

Infant posture and spontaneous motility hold significance in early development [1]. According to Pretchtl's study [2], the spontaneous movements, known as general movements (GMs), has been approved, and an assessment method that can be clinically used at the first 5 months post-term age (PTA) was developed. General movements assessment (GMA) is an observation assessment used worldwide and can serve as a predictive indicator particularly of

fidgety movements (FMs, small-moderate speed movement, and varying acceleration in all directions across the neck, trunk, and limbs) for identifying the occurrence of neurodevelopment problems (e.g. cerebral palsy, CP) or movement disorders [3–6]. Based on GMA, a tool, the movement optimality scale (MOS) [2,7], was developed, which considers postural and movement patterns in detail, for instance, the position of the head, body, and limbs, asymmetric tonic neck posture (ATNP), and limb movements including movement toward midline (MTM, i.e. foot-to-foot contact, fiddling, hand-to-hand and hand-to-mouth contact). This tool's majority score is the presence of FMs and calculates the score of posture and movement subgroups altogether. The result can range from 5 to 28, which indicates high-risk precautions for scores <19 [8,9].

MTM is characterised by limb activities moving against gravity and close to the imagination midline which symmetrically divides the body into the left and right. In early infancy, this motor pattern was considered as initially developmental toward intentional movement behaviour and oriented toward specific objectives. Lucaccioni et al. [10] expanded the understanding of the typical and age-adequate motor repertoire regarding MTM of four limbs by observing and identifying the developmental progression in healthy, full-term infants from birth until 22–23 weeks PTA. Infants were observed to have 10 MTM including foot-to-foot (FF), foot-to-leg (FL), hand-to-hand (HH), hand-to-face (HF), hand-to-mouth (HM), hand-to-trunk (HT), hand-to-leg (HL), manipulation, pedipulation, and fiddling in writhing and fidgety movement periods. Progressive increases in frequencies were notably observed, particularly during the age of FMs. Ferrari et al. [11] examined the occurrence of FMs and FF in 58 full-term infants diagnosed with hypoxic-ischemic encephalopathy who underwent brain cooling treatment. The study categorised the cases into four sub-groups: infants with both FMs and FF present, those with present FMs but absent FF, those with absent FMs but present FF, and those with both FMs and FF absent. Within a subgroup consisting of 18 cases with the

absence of both FMs and FF, 3 cases were observed to subsequently develop CP. The findings of this study propose that FF represents a straightforward and easily evaluable motor pattern. Moreover, it could enhance the sensitivity and specificity of FMs. Incorporating the assessment of goal-directed movements may serve as a supplementary assessment in early-age infants.

Previous studies [12–14] demonstrated various movement assessment methods, including visual observation, wearable sensor technology systems, and markerless systems for infant movement monitoring. Utilising specialised techniques, such as three-dimensional motion capture with wearable sensors, to obtain quantitative data can result in high production costs, requiring a specific setting, and demand expertise [15]. For instance, Kanemaru et al. [16] conducted a study using the motion analysis system to investigate the end-point movement focusing on individual limbs and interlimb coordination in 2- to 4-month-old healthy infants and analysed the indices to be a precursor for goal-directed movement. Miyagishima et al. [17] performed a comparison between preterm infants (corrected age, 1–3 months) and term infants, utilising sensors and a 3D motion capture system to analyse the characteristics of antigravity spontaneous movements which encompassed measurements of distance and height for both hands and feet. Marchi et al. [18] examined the predictive capability of quantitative computerised kinematic indices in a group of 12 low-risk term infants processing by motion analysis system with 6 markers. The analysis was done by interpreting indexes of coordination, distance, and global movement quality based on the infants' motions toward the midline. Two-dimensional (2D) pose estimation is an alternative computer vision technique used to estimate the pose of a person or object in a 2D image or video [19,20]. This is a real-time multi-person detection method based on the x and y coordinates of human body joints from RGB (red, green, and blue) images [21]. Pose estimation has been applied to diverse fields, such as sports, home care, work monitoring, and surveillance [22]. The study of marker-free infant movement has

shown advantages in which infants perform actions naturally with minimal cost, less time consumption, and measurable quantitative data [23–25].

Movement analysis exhibits a wide range of diversity, and its level of accessibility depends on numerous elements, encompassing the assessor, the client, available resources, locations, technological advancements, budgetary considerations, and other relevant factors. While infants show movements directed toward the midline in a 2D image or video, an observable reduction in the distance between the distal part of limbs and body midline is possible. Furthermore, when measuring the spatial area defined by the endpoint of the extremity and midpoint of the body, deviations from this midpoint close to the midline may lead to a reduction in the enclosed area when compared to instances where the limbs are positioned farther from the midpoint. Therefore, this study focused on using video-based assessment with a pose estimation analysis technique on datasets of young term-infant movements and extracting features concerning MTM. We aimed to identify the correlation between features from pose estimation data, for example, distance and area, and infant movements regarding MTM, in an early-age infant (i.e. 8–16 weeks PTA). Prospectively, the coordination data could help develop an index to quantify infant movements or behaviours in the first few months of life, supporting other movement assessments and allowing easy monitoring, particularly in low-resource settings.

Materials and Methods

Participants

This study collected data using video recordings from 20 infants aged 8–16 weeks PTA. Gestation age (GA) was corrected for the term birth (40 weeks + 0 days). We reported birth weight, birth length, birth head circumference with a range of percentile following WHO child

growth standards, 2006 [26]. Moreover, the Apgar scores at min 1 and 5 were presented (S1 Table). The recruitment period for this study started from 24/03/2022 to 28/02/2023. Participant recruitment was performed using two methods: direct contact with parents/guardians taking care of young infants and asking if they wished to participate in this research voluntarily via email or in person or via poster invitations in parent support groups on social media asking for voluntary participation. At the onset, the study details were conveyed individually to each parent or guardian. Comprehensive information sheets were provided, and they were actively encouraged to seek clarification. Upon gaining a thorough understanding of the process, they proceeded to electronically complete a consent form, specifying the participant's name, their relationship, the signatures of the parents or guardians, and the date. Then, we asked parents or guardians to send a video of their infants through their mobile phones using a private mobile application, Dropbox (online cloud storage). The inclusion criteria were infants without any diagnosed diseases or abnormalities, and the selected videos were good-quality 2-minute recordings of active infants lying supine, excluding crying or sleeping during the video. Videos were excluded if data extraction using 2D pose estimation was not possible. This study was approved by the relevant human research ethics committee (no. 21092).

Video recording and collecting procedure

Parents and guardians were instructed to record videos of the infants by placing them on a firm mat in the supine position. Infants were asked to wear a nappy and a bodysuit. The mobile camera was held on the top of the infants, approximately 1 m away (top view covering all limbs of the infant). Parents or guardians recorded a video for 2–3 min and avoided interacting during the recording. Afterwards, the video file was sent to the researcher via Dropbox. This service provides multiple security settings for sharing sensitive information and aims to prevent third parties from accessing the data. First, the researcher created a personal

folder with a password that only the researcher and parents/guardians could access. Then, the parents/guardians were educated on installing and accessing the application and setting password protection on video files before uploading them. After the video was saved in a locked USB storage, the researcher immediately removed the video files from the application. Dropbox sends notifications of changes to those involved in the files/folder. After collecting the videos from the infants' parents/guardians, the length of the videos was assessed and cut into 2 min. No specific instructions were given to the guardians regarding when to start recording. Consequently, each video commenced by pressing the recording button. The initial 5 s of the video were cut off and subsequently maintained for 2 mins thereafter.

Procedure of MTM occurrence observation

All included videos were manually observed and scored as absent or present when infants showed MTM during the 2-min video, defined as following [6]: FF elevating both legs, the feet are occasionally brought together with the soles touching each other for a second; FL showing both legs are elevated, and the sole of one foot touches the opposite leg for a second; HH showing both hands are brought together and contact each other in the midline for a second; HF showing one hand or both hands reach and contact the face in the midline for a second; HM showing one hand or both hands reach the lip with the head aligned at the midline and the fingers may or may not enter the mouth for a second; HT showing one hand or both hands reach and touch any part of the trunk for a second; HL showing the hand reaches and touches the leg or knee on the same side for a second; FF pedipulation showing feet sliding or grabbing, ensure that the feet remain in contact at the midline and the legs are elevated for a second; HH manipulation showing both hands lift antigravity and are brought together at the midline with sliding and grabbing movements for a second; and fiddling, showing one or both hands repeatedly touch, stroke, or grasp an object, often own clothing, for a second. The description

of each MTM is clear and easy to follow, thus simplifying the observation process. In addition, the risk of erroneously counting the appearance of an MTM when it was absent was minimised.

According to the definition, MTM was recorded when infants exhibited MTM lasting for at least 1 second. Firstly, the 2-min video was cut down to 1-s intervals with 120 video files for each video. The observer then prepared a record form for 10 MTM and each video interval according to the second unit (120 checkboxes) in an Excel 2016 spreadsheet. The observer went through 120 s and gave one remark in case the infant showed any MTM persisting for a second. Subsequently, we calculated the occurrence of MTM for each total time interval and then aggregated the results by summing the occurrences of MTM over 10 intervals to obtain the overall count. The MTM occurrence percentage was calculated by dividing the total MTM occurrence time by 120 (video duration) and multiplying by 100. Additionally, the MTM occurrence rate per minute was calculated, which was divided into three groups: total MTM occurrence rate per minute, lower-limb MTM occurrence rate per minute (FF, FL, and pedipulation), and upper-limb MTM occurrence rate per minute (HH, HF, HM, HT, HL, manipulation and fiddling) [27]. This was computed by dividing the total duration of MTM occurrences or the duration of lower or upper limb MTM by the length of the video in seconds and then multiplying the result by 60.

Procedure of pose estimation and getting coordinates (x,y)

In this study, we processed 2D pose estimation using MediaPipe [22] to extract 33 body landmark coordinates (x, y) from each 2-min video. The total number of frames was 3,600, with a frame width and height of 1920 × 1080 and 118, 800 coordinates. Python was applied using PyCharm. After running the code, we obtained all the coordinate values in comma-separated value (csv) files. We then used all the landmark coordinates to determine the centre of the body coordinates. The selection of wrist and ankle coordinates (landmarks 15 and 16 and

27 and 28, respectively) was utilised to calculate the distance between the limbs and imaginary midbody line, as well as the areas. The landmarks were computed using Mediapipe, utilising normalised screen coordinates obtained from the x and y coordinates. The distances and areas were normalised based on the average left- and right-body distances. Consequently, these points yielded indices with dimensionless values.

Procedure for finding the distance between limb landmarks and the mid-body imaginary line

We obtained the limb to midline distance using Python coding in a Jupyter Notebook (anaconda3) csv file. First, we determined the midpoint of the shoulder and hip, landmarks 11–12 and 23–24, respectively, based on the midpoint formula $(x_m, y_m) = (\frac{x_1 + x_2}{2}, \frac{y_1 + y_2}{2})$. Then, to find the imaginary line, we applied both midpoint (x, y) values to the line equation y = mx + c, substituting the y value of limb landmark coordinates (15 and 16 for the wrists, 27 and 28 for the ankles) to obtain the x value at the imaginary line, followed by subtracting x of the landmarks to obtain the distance (Fig 1). All distance values were normalised to the average of the left and right body lengths (Euclidean distances of landmarks 11–23, 12–24). After obtaining the distance values from all the frames, we calculated the average distance of each of the four landmarks from the midline. In addition, the distance of landmark (Dlm) 15 represents the distance between landmark 15 and the imaginary line, Dlm16 represents the distance between landmark 16 and the imaginary line, and so on for Dlm27 and Dlm28.

Fig 1. Pose landmark [28] **and calculated distance.** This figure shows limbs' distance (dash line, ---) from the number of landmarks 15, 16, 27, and 28 to the midbody imaginary line.

Procedure for finding body areas

In this study, we focused on the entire body and the upper- and lower-limb areas (Fig 2) using wrist and ankle landmarks (15 and 16 and 27 and 28, respectively) to calculate the quadrilateral area representing the entire area. In addition, we determined the coordinates of the body centre, as shown in Figure 2B, by calculating the centroid using all 33 landmarks, then finding the triangular area for the upper part using landmarks 15 and 16 and the centre of the body coordinate, as well as the lower part using landmarks 27 and 28 and the centre of the body coordinates. All the area values were normalised to the average of the left and right body lengths, which were the same as the distance. Each video consisted of 3,600 measurements for each area value, and the statistical analysis employed the average values derived from each video.

Fig 2. Pose landmark [28] **and calculated area.** This figure shows calculated area using limbs pose landmarks (15, 16, 27, and 28) and centre of the body coordinate which are listed as: (**A**) presenting the whole area; (**B**) presenting ❶ = the upper part area, and ❷ = the lower part area.

Data analysis

In this study, the values from the MTM observations were as follows: MTM occurrence percentage, MTM occurrence rate per minute (total, lower limb, and upper limb), and pose estimations features were distance and area. Statistical analyses were performed using SPSS for Windows version 26 (Armonk, NY: IBM Corp.). Normality was assessed using the Kolmogorov–Smirnov Goodness of Fit test. The results were non-normally distributed except for whole area that showed normal distribution. The Spearman correlation test was used to

calculate the correlation coefficient (r) between the average distance of the four landmarks to midbody imagination line and MTM observation values.

Then, we categorised the videos into two distinct groups: one featuring MTM and the other without MTM. Within these groups, we further classified the videos into three categories: those displaying Lower MTM, Upper MTM, and a combination of both, which we collectively labelled as 'Total MTM.' We employed an independent t-test to compare the whole area, whereas the Mann–Whitney U test was utilised to compare the upper- and lower-part areas. Statistical significance was defined as $p < 0.05$. The overall study methodology is depicted in Fig 3.

Fig 3. Flow chart of the study methodology. MTM: movement toward midline

Results

A total of 98 videos (20 infants, aged 8–16 weeks PTA) were recruited for this study; 4 videos were excluded because of errors in data extraction. Therefore, 94 infant videos were included in this study. Anthropometric data is summarised in Table 1. The distribution of 10 MTM items were presented, with HT and FF contacts being the most frequently observed in the upper- and lower-limb MTM, respectively (Fig 4).

Table 1. Anthropometric data at birth according to WHO Growth Standard for children 0-2 and 2-5 years of age, 2006 [26]

	**Gestational age (weeks + days)**	**Weight (g)**	**Length (cm)**	**Head circumference (cm)**
**Median** (range of percentile)	38 + 4	3150 (P25-P50)	49 (P25-P50)	34 (P25-P50)
**Min : Max** (range of percentile)	37 : 40 + 1<br>-	2550 : 3900<br>(P3-P10) : (P90-P97)	47 : 55<br>(P10-P25) : (>P97)	31 : 36.5<br>(<P3) : (P90-P97)

g = grams, cm = centimetres

Fig 4. The distribution of MTM frequency among infants who showed MTM on the recording day. This figure shows the data that was observed MTM occurrence based on infant age in days post-term age (PTA). MTM: movement toward midline

Correlation between the calculated distance between limb landmarks and MTM observation

In this study, we investigated the relationship between the distance from upper limb landmarks to the midbody imagination line (Dlm15 for the left side and Dlm16 for the right side) and the MTM occurrence. Our findings showed that there were significant weak correlations between these distances and the total MTM occurrence percentage (r_{Dlm15} = 0.315, r_{Dlm16} = -0.354), as well as the occurrence rate per minute for upper-limb MTM (r_{Dlm15} = 0.372, r_{Dlm16} = -0.393) and total MTM (r_{Dlm15} = 0.319, r_{Dlm16} = -0.360). Considering the distance for lower limb landmarks to the midbody imagination line (Dlm27 for the left side and Dlm28 for the right side) and the MTM occurrence, the distance from the right ankle landmark (Dlm28) showed significantly weak correlations to total MTM occurrence percentage (r_{Dlm28} = -0.331), the occurrence rate per minute for lower-limb MTM (r_{Dlm28} = -0.354), and total MTM (r_{Dlm28} = -0.334) (Table 2).

Table 2. Correlation between distances from four limb landmarks to midbody imaginary line and MTM observation

	Dlm15	Dlm16	Dlm27	Dlm28
Total MTM Occurrence percentage	**0.315*** (0.12, 0.4866)	**-0.354*** (-0.519, -0.163)	-0.103 (-0.299, 0.1017)	**-0.331*** (-0.5, -0.138)
Lower MTM occurrence rate per minute	0.027 (-0.177, 0.2284)	-0.007 (-0.209, 0.1959)	-0.199 (-0.386, 0.0038)	**-0.354*** (-0.519, -0.163)
Upper MTM occurrence rate per minute	**0.372*** (0.1832, 0.5343)	**-0.393*** (-0.552, -0.207)	-0.009 (-0.211, 0.194)	-0.211* (-0.397, -0.009)
Total MTM occurrence rate per minute	**0.319*** (0.1244, 0.4899)	**-0.360*** (-0.524, -0.17)	-0.095 (-0.292, 0.1097)	**-0.334*** (-0.503, -0.141)

This table reported correlation coefficient (r) and its corresponding 95% confidence interval in parentheses. MTM: movement toward midline, * p-value < 0.01

Comparison of video groups that show MTM and no MTM

In the dataset, we found the following distribution of videos across three categories: Total MTM (82 videos showed MTM; 12 did not), Lower MTM (36 videos showed MTM; 58 did not), and Upper MTM (76 videos showed MTM; 18 did not). Significant differences were detected in both the whole area ($p = 0.001$) and lower part area ($p = 0.003$) within the Lower MTM group. However, no significant differences were found in area values observed for the Upper MTM and Total MTM groups (Fig 5).

Fig 5. The difference between groups of videos that showed MTM and did not show MTM, comparing the calculated whole area, upper part, and lower part area which categorised into three groups of MTM: Lower MTM, Upper MTM, and Total MTM. MTM: movement toward midline, * p-value < 0.05.

Discussion

In this study, we collected videos of healthy infants aged 8–16 weeks PTA (recording by parents or guardians) and observed the occurrence of MTM within 10 MTM (Lower MTM included FF, FL, and pedipulation, whereas Upper MTM encompassed HH, HF, HM, HT, HL, manipulation, and fiddling). We then utilised 2D pose estimation to extract landmark coordinates, enabling the calculation of distances and areas. There were three major findings of this study: in the upper MTM, 'HT' was the most frequently observed motor behaviour, whereas in the lower MTM, 'FF' was the most prevalent; we identified weak correlations between the calculated distance and MTM occurrence values; and significant differences were observed in both the whole area and lower part area between videos showing MTM and those without MTM within the Lower MTM category.

MTM was considered the initial antigravity movement behaviour that would eventually develop into goal-directed movements. In this study, the FF and HT movement patterns were most frequently observed for the lower and upper limbs, respectively. In typical infants, lower limb movement normally begins with leg lifting and then progresses to foot contact and exploration with various patterns of leg movements [29,30]. FF movement serves as an early antigravity movement, potentially identifying neurodevelopmental problems during early infancy. It can be effectively used in conjunction with fidgety movement assessments as a precursor movement measurement [11]. HT is a fundamental motor behaviour observed in infants when they exhibit fewer uncontrolled movements (i.e. swipes and oscillations) or it could be from ANTP. They bring their arms and hands to contact their body and initiate self-exploration. This motor behaviour can enhance somatosensory input, skill development, and subsequent behaviours [31,32].

Considering the correlation between the distance calculated from upper arm landmarks and MTM, occurrence values revealed significant positive correlations with left wrist

landmarks and negative correlations with right wrist landmarks. These associations may be attributed to the infant's posture, where one hand contacts the body while the other remains apart. The presence of ATNP may influence this outcome, as ATNP occurs when the infant rotates their head to the side while extending one arm and leg and flexing the arm and leg on the opposite side [10,33].

In the lower-limb MTM group, significant differences were observed in the whole area and lower area between videos that showed MTM and no MTM. In this study, the lower limb MTM category encompassed FF, FL, and pedipulation movements. These specific movements involve positioning the ankle and foot closer to each other, resulting in a smaller area compared with having the legs apart. Infants require strength, skill, and experience to bring their feet together while lifting their legs [34,35]. This motor behaviour may lead to intentional goal-directed movement [34,36,37].

In the upper-limb MTM group, no significant difference was found in all areas between video showing MTM and no MTM. This category encompasses various wrist positions, including both wrists together and one wrist apart, which can contribute to varying areas of the upper trunk. Further investigations could involve grouping arm positions and comparing corresponding areas to gain a more comprehensive understanding of the subject matter. In addition, postural control may affect upper-limb MTM, especially HH movements, in the supine position [38,39]. Infants may require body stabilisation before they can achieve smooth limb movements to ensure maintenance and optimal functioning.

The study limitations were as follows: video recording was done by a parent or guardian. It was common to have some interferences that cannot be avoided from the presence of them while approaching the infant at this age; despite efforts to maintain a perpendicular position for handheld smartphones during recording, slight movements or changes in camera angles owing to the camera-holding hand were possible. However, since the features calculated

in this study are angular information obtained by connecting markers with lines and distance information corrected by body length, we believe that their influence was minimized. In the future, it is necessary to obtain more stable and reliable data by fixing the camera.

Conclusions

We observed a significant correlation between MTM observation values and distances calculated from limb landmarks to the midbody imaginary line. Notably, both the whole area and the lower part area exhibited significantly lower values in the videos showing MTM, especially in the Lower MTM group. The 2D pose estimation features (coordinates, distance, and area) can be utilised to provide support in identifying the presence of MTM in infants. Infant video recordings were captured by parents/guardians. This method offers advantages in cases where regular follow-up is challenging, particularly in isolated areas or situations with a risk of infection. Furthermore, analysing video data with a computer program enabled natural infant movement. A significant association between MTM observation values and distance or area was observed. Hence, the development of a dedicated 2D pose estimation system for infants is essential to improve accuracy and accommodate the variety of MTM. Additionally, conducting MTM assessments to monitor later developmental skills and gather data from at-risk infants is crucial.

Acknowledgments

We extend our heartfelt gratitude to Dr. Nuttawat Rungsirisilp and Dr. Kornkanok Tripanpitak for their invaluable support during the pose estimation and feature extraction processes.

References

1. Cioni G, Ferrari F, Prechtl HFR. Posture and spontaneous motility in fullterm infants. Early Hum Dev. 1989;18: 247–262. doi:10.1016/0378-3782(89)90020-0
2. Einspieler C, Prechtl HFR, Bos A, Ferrari F, Cioni G. Prechtl's method on the qualitative assessment of general movements in preterm, term and young infants. Clin Dev Med. 2004;167: 1–91.
3. Cioni G, Prechtl HF. Preterm and early postterm motor behaviour in low-risk premature infants. Early Hum Dev. 1990;23: 159–191. doi:10.1016/0378-3782(90)90012-8
4. Einspieler C, Prechtl HFR. Prechtl's assessment of general movements: A diagnostic tool for the functional assessment of the young nervous system. Ment Retard Dev Disabil Res Rev. 2005;11: 61–67. doi:10.1002/mrdd.20051
5. Novak I, Morgan C, Adde L, Blackman J, Boyd RN, Brunstrom-Hernandez J, et al. Early, accurate diagnosis and early intervention in cerebral palsy: Advances in diagnosis and treatment. JAMA Pediatr. 2017;171: 897–907. doi:10.1001/jamapediatrics.2017.1689
6. Einspieler C, Marschik PB, Bos AF, Ferrari F, Cioni G, Prechtl HF. Early markers for cerebral palsy: Insights from the assessment of general movements. Future Neurol. 2012;7: 709–717. doi:10.2217/fnl.12.60
7. Einspieler C, Bos AF, Krieber-Tomantschger M, Alvarado E, Barbosa VM, Bertoncelli N, et al. Cerebral palsy: Early markers of clinical phenotype and functional outcome. J Clin Med. 2019;8: 1616. doi:10.3390/jcm8101616
8. Örtqvist M, Einspieler C, Marschik PB, Ådén U. Movements and posture in infants born extremely preterm in comparison to term-born controls. Early Hum Dev. 2021;154: 105304. doi:10.1016/j.earlhumdev.2020.105304
9. Peyton C, Millman R, Rodriguez S, Boswell L, Naber M, Spittle A, et al. Motor optimality scores are significantly lower in a population of high-risk infants than in infants born

moderate-late preterm. Early Hum Dev. 2022;174: 105684. doi:10.1016/j.earlhumdev.2022.105684.

10. Lucaccioni L, Bertoncelli N, Comini M, Martignoni L, Coscia A, Lugli L, et al. The ontogeny of limbs movements towards midline in healthy infants born at term. Early Hum Dev. 2021;155: 105324. doi:10.1016/j.earlhumdev.2021.105324
11. Ferrari F, Bedetti L, Bertoncelli N, Roversi MF, Della Casa E, Guidotti I, et al. Foot-to-foot contact among initial goal-directed movements supports the prognostic value of fidgety movements in HIE-cooled infants. Front Pediatr. 2022;9. doi:10.3389/fped.2021.731021
12. Silva N, Zhang D, Kulvicius T, Gail A, Barreiros C, Lindstaedt S, et al. The future of general movement assessment: The role of computer vision and machine learning – A scoping review. Res Dev Disabil. 2021;110: 103854. doi:10.1016/j.ridd.2021.103854
13. Chen H, Xue M, Mei Z, Bambang Oetomo S, Chen W. A review of wearable sensor systems for monitoring body movements of neonates. Sensors (Basel). 2016;16: 2134. doi:10.3390/s16122134
14. Irshad MT, Nisar MA, Gouverneur P, Rapp M, Grzegorzek M. AI approaches towards Prechtl's assessment of general movements: A systematic literature review. Sensors (Basel). 2020;20. doi:10.3390/s20185321
15. Stenum J, Rossi C, Roemmich RT. Two-dimensional video-based analysis of human gait using pose estimation. PLOS Comp Biol. 2021;17: e1008935. doi:10.1371/journal.pcbi.1008935
16. Kanemaru N, Watanabe H, Taga G. Increasing selectivity of interlimb coordination during spontaneous movements in 2- to 4-month-old infants. Exp Brain Res. 2012;218: 49–61. doi:10.1007/s00221-012-3001-3

17. Miyagishima S, Asaka T, Kamatsuka K, Kozuka N, Kobayashi M, Igarashi R, et al. Characteristics of antigravity spontaneous movements in preterm infants up to 3 months of corrected age. Infant Behav Dev. 2016;44: 227–239. doi:10.1016/j.infbeh.2016.07.006
18. Marchi V, Belmonti V, Cecchi F, Coluccini M, Ghirri P, Grassi A, et al. Movement analysis in early infancy: Towards a motion biomarker of age. Early Hum Dev. 2020;142: 104942. doi:10.1016/j.earlhumdev.2019.104942
19. Stenum J, Cherry-Allen KM, Pyles CO, Reetzke RD, Vignos MF, Roemmich RT. Applications of pose estimation in human health and performance across the lifespan. Sensors (Basel). 2021;21: 7315. doi:10.3390/s21217315
20. Sermpon N, Gima H. A systematic review of the measurement of infant posture and movement using image or video data analysis | Archives of allied health sciences. Arch AHS. 2022;34: 1–15.
21. Cao Z, Hidalgo G, Simon T, Wei S-E, Sheikh Y. OpenPose: Realtime multi-person 2D pose estimation using part affinity fields. IEEE Trans Pattern Anal Mach Intell. 2021;43: 172–186. doi:10.1109/TPAMI.2019.2929257
22. Kim J-W, Choi J-Y, Ha E-J, Choi J-H. Human pose estimation using MediaPipe pose and optimization method based on a humanoid model. Appl Sci. 2023;13: 2700. doi:10.3390/app13042700
23. Hesse N, Sebastian Schroeder A, Müller-Felber W, Bodensteiner C, Arens M, G. Hofmann U. Markerless motion analysis for early detection of infantile movement disorders. In: Eskola H, Väisänen O, Viik J, Hyttinen J, editors. EMBEC & NBC 2017. Singapore: Springer; 2018. pp. 197–200. doi:10.1007/978-981-10-5122-7_50
24. Reich S, Zhang D, Kulvicius T, Bölte S, Nielsen-Saines K, Pokorny FB, et al. Novel AI driven approach to classify infant motor functions. Sci Rep. 2021;11: 1–13. doi:10.1038/s41598-021-89347-5

25. Chambers C, Seethapathi N, Saluja R, Loeb H, Pierce SR, Bogen DK, et al. Computer vision to automatically assess infant neuromotor risk. IEEE Transactions on Neural Systems and Rehabilitation Engineering, Neural Systems and Rehabilitation Engineering, IEEE Transactions on, IEEE Trans Neural Syst Rehabil Eng. 2020;28: 2431–2442. doi:10.1109/TNSRE.2020.3029121

26. The WHO child growth standards. [cited 5 Sep 2023]. Available: https://www.who.int/tools/child-growth-standards

27. Sermpon N, Gima H. Relationship between fidgety movement and frequency of movement toward midline: An observational study. Early Hum Dev. 2023;177–178: 105718. doi:10.1016/j.earlhumdev.2023.105718

28. Pose landmark detection guide | MediaPipe. In: Google for Developers [Internet]. [cited 6 Sep 2023]. Available: https://developers.google.com/mediapipe/solutions/vision/pose_landmarker

29. Piek JP, Carman R. Developmental profiles of spontaneous movements in infants. Early Hum Dev. 1994;39: 109–126. doi:10.1016/0378-3782(94)90160-0

30. Deng W, Vanderbilt DL, Smith BA. Differences in spontaneous leg movement patterns between infants with typical development and infants at risk for developmental delay: Cross-sectional observation prior to sitting onset. J Mot Learn Dev. 2018;6: 101–113. doi:10.1123/jmld.2016-0056

31. Babik I, Galloway JC, Lobo MA. Infants born preterm demonstrate impaired exploration of their bodies and surfaces throughout the first 2 years of life. Phys Ther. 2017;97: 915–925. doi:10.1093/ptj/pzx064

32. Kanazawa H, Yamada Y, Tanaka K, Kawai M, Niwa F, Iwanaga K, et al. Open-ended movements structure sensorimotor information in early human development. Proc Natl Acad Sci U S A. 2023;120: e2209953120. doi:10.1073/pnas.2209953120

33. Hamer EG, Hadders-Algra M. Prognostic significance of neurological signs in high-risk infants – A systematic review. Dev Med Child Neurol. 2016;58: 53–60. doi:10.1111/dmcn.13051
34. Galloway JC, Thelen E. Feet first: Object exploration in young infants. Infant Behav Dev. 2004;27: 107–112. doi:10.1016/j.infbeh.2003.06.001
35. Discovery of motor development: A tribute to Esther Thelen. [cited 19 Jun 2023]. Available: https://psycnet.apa.org/fulltext/2014-44019-007.html
36. Kim JA, Fetters L, Kubo M, Eckel SP, Sargent B. Infants born full term and preterm increase the height of anti-gravity leg movements during a kick-activated mobile task using a scaffolded task environment. Infancy. 2021;26: 168–183. doi:10.1111/infa.12379
37. Heathcock JC, Galloway JC. Exploring objects with feet advances movement in infants born preterm: A randomized controlled trial. Phys Ther. 2009;89: 1027–1038. doi:10.2522/ptj.20080278
38. Rocha NACF, Tudella E. The influence of lying positions and postural control on hand–mouth and hand–hand behaviors in 0–4-month-old infants. Infant Behav Dev. 2008;31: 107–114. doi:10.1016/j.infbeh.2007.07.004
39. Dusing SC, Harbourne RT. Variability in postural control during infancy: Implications for development, assessment, and intervention. Phys Ther. 2010;90: 1838–1849. doi:10.2522/ptj.2010033

Supporting information

S1 Table Infant demographic data (N = 20) This table included gestational age (GA) and anthropometrics at birth. Percentiles are presented according to WHO Growth Standard for children 0–2 and 2–5 years of age, 2006 [26]. The Apgar scores at one and five minutes, along with the age at the time of video collection, are provided for each infant.

Figure 1

Click here to access/download;Figure;Fig 1.tif

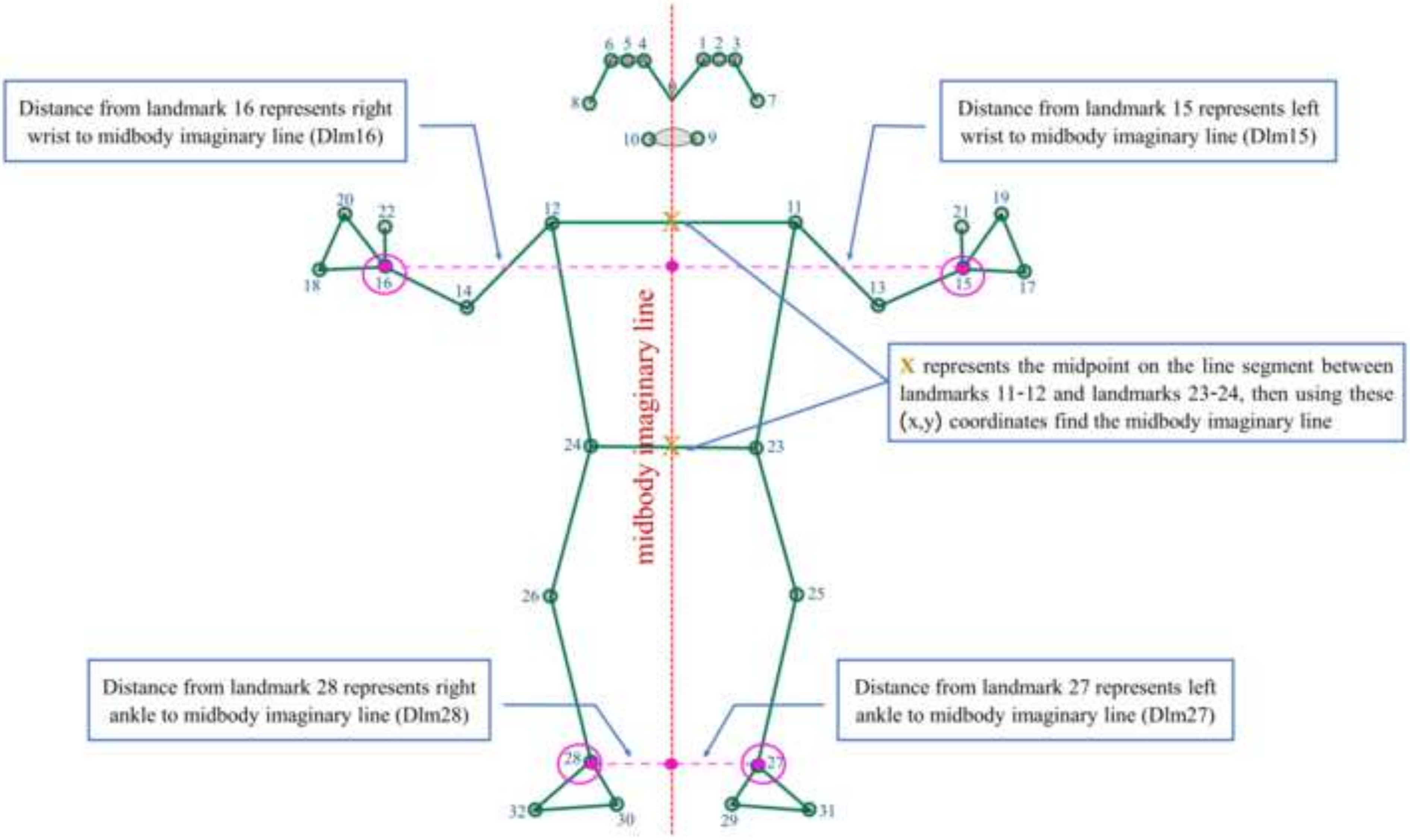

Figure 2

Click here to access/download;Figure;Fig 2.tif

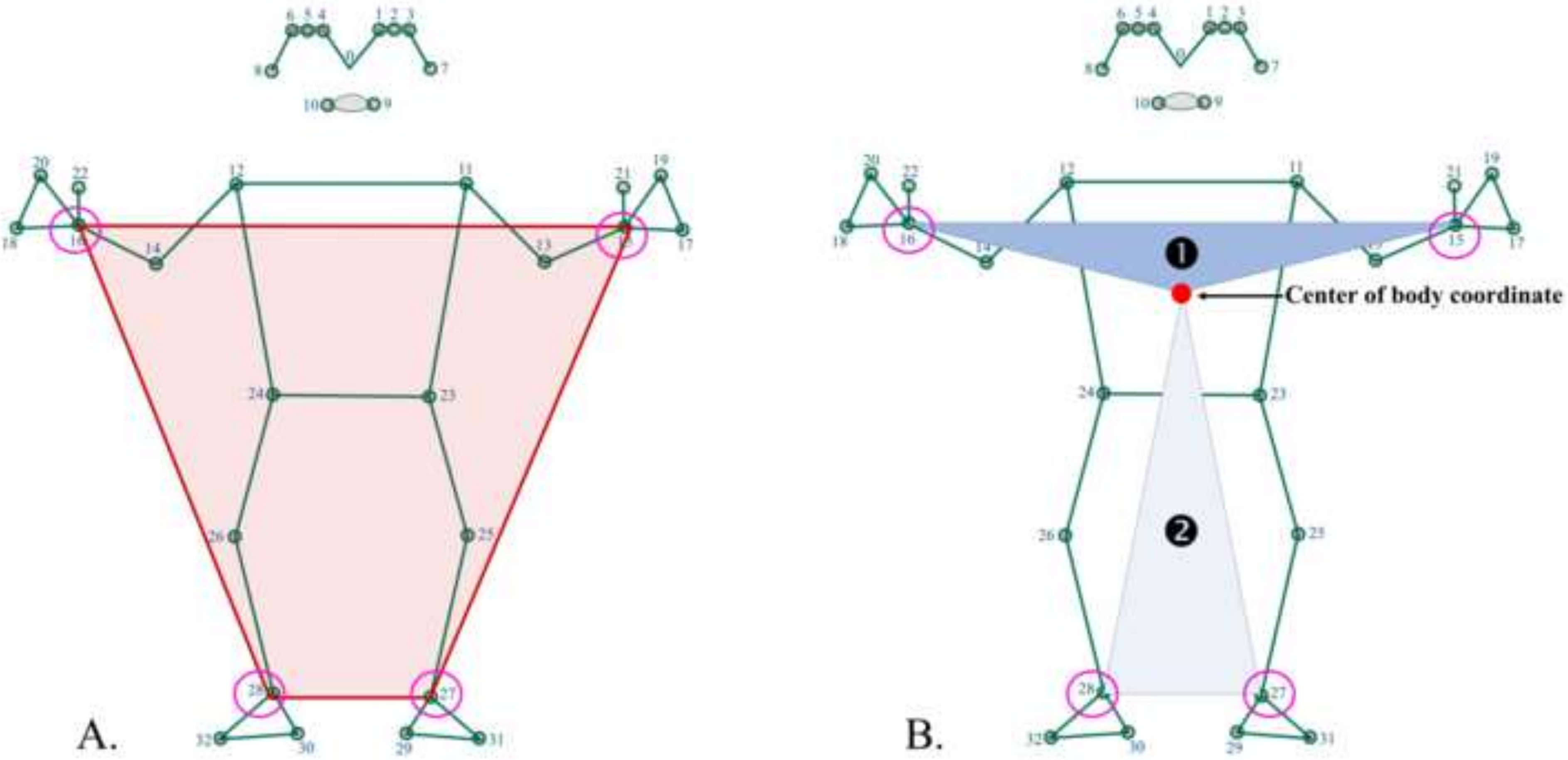

 Click here to access/download;Figure;Fig 3.tif

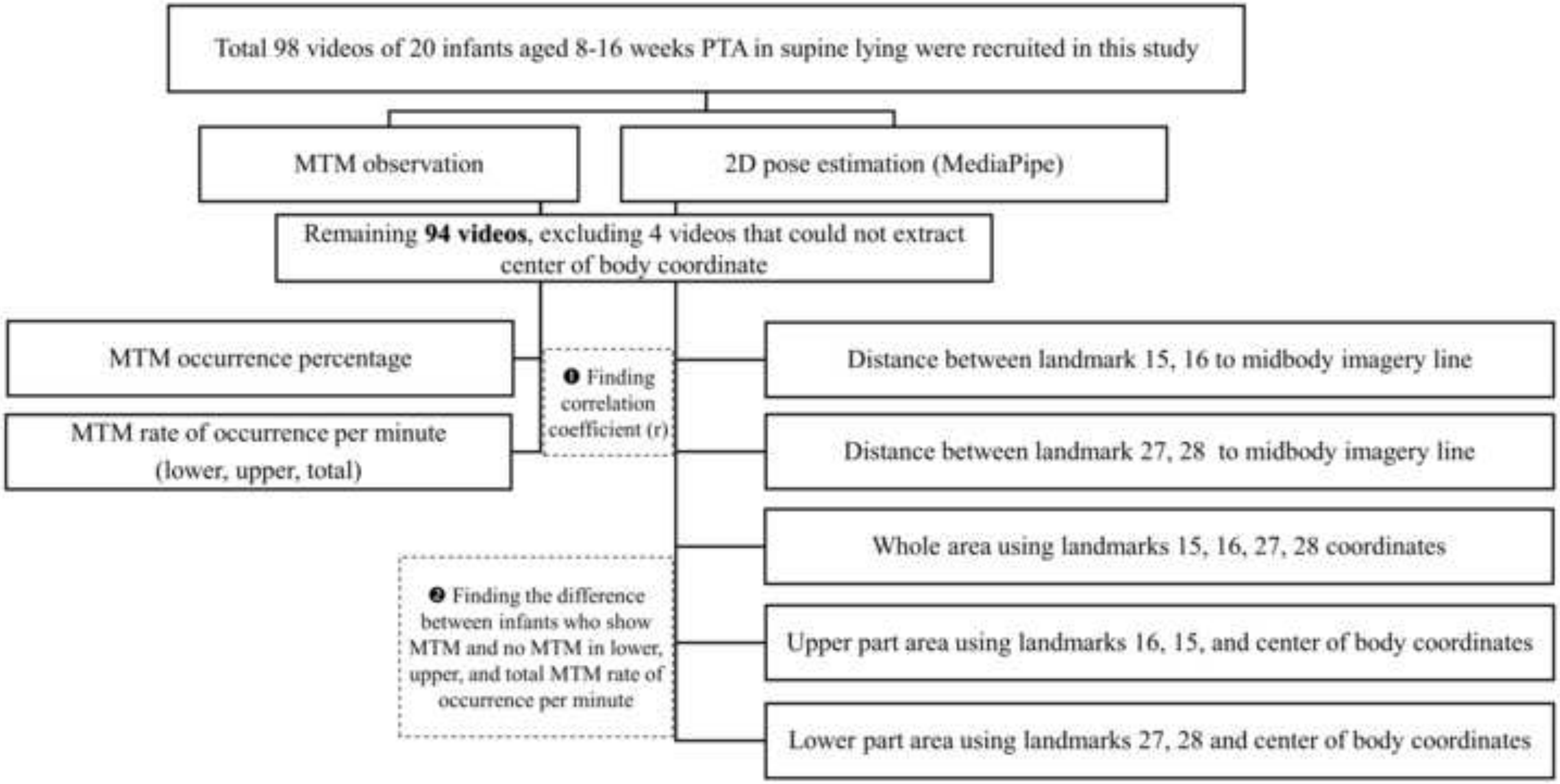

Click here to access/download;Figure;Fig 4.tif

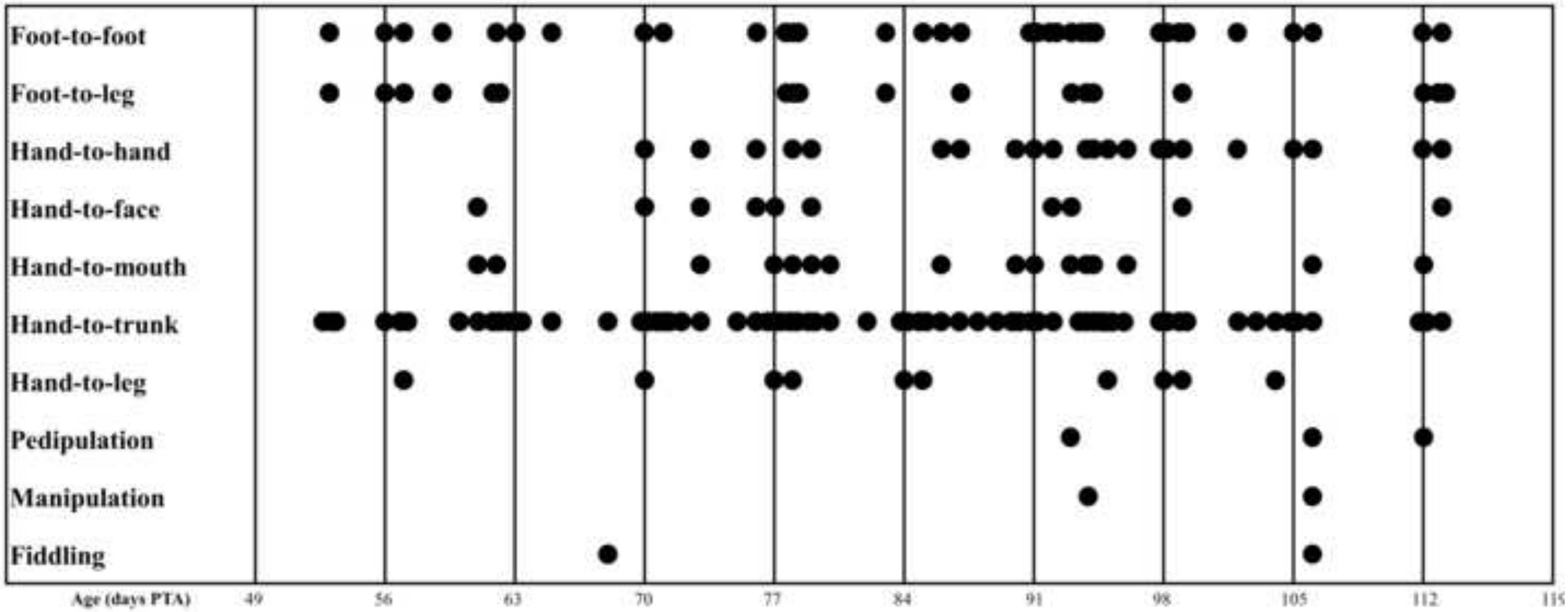

Figure 5

Click here to access/download;Figure;Fig 5.tif

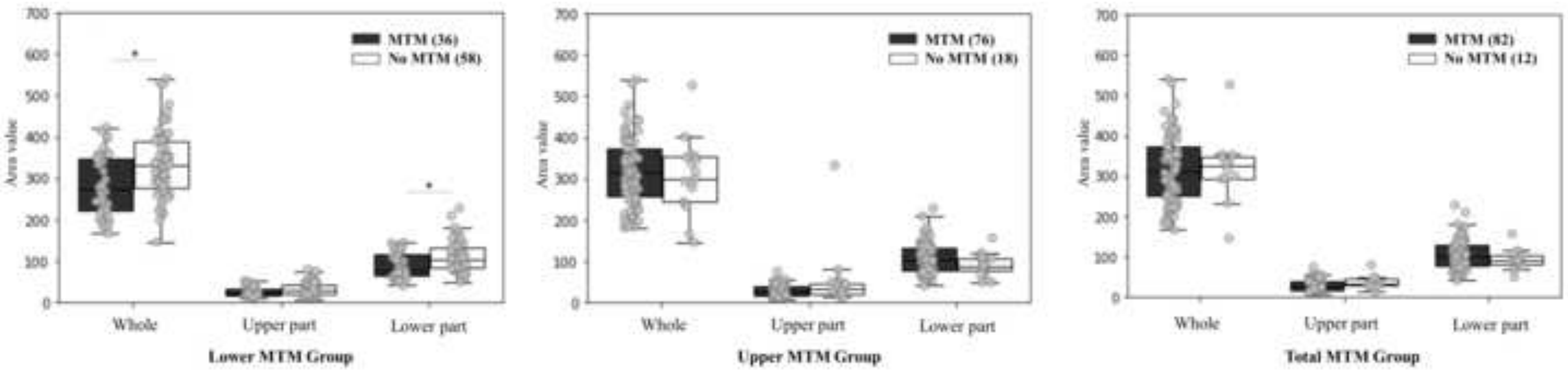

Supporting Information 1 Table

Click here to access/download

Supporting Information

003_S1 Table Infant demographic data.docx
