# Supplementary material for: Correlation between pose estimation features regarding movements towards the midline in early infancy
Source: PLoS One. 2024 Feb 28;19(2):e0299758. doi: 10.1371/journal.pone.0299758 (PMC10901309; doi:10.1371/journal.pone.0299758)
Supplement: Attachment — Submitted filename: PONE-D-23-29999 Summary Review.docx [file pone.0299758.s002.docx]

Dear Dr. Gima,

Many thanks to you and Dr. Sermpon for your work exploring the application of video pose estimation to quantify movement toward the midline (MTM) in young infants. I enjoyed reading your manuscript and learned from it. Overall, it is well-written and it is obvious that your study was thoughtfully designed and carried out with care. I have summarized my impressions below and highlight areas where more clarity may be desirable. I annotated the PDF of your study as I read it. I am including that for your consideration as well.

Most sincerely,

Manon Maitland Schladen

**Summary of Research and Overall Impression**

This study reports the feasibility of using an open source, pose estimation model (MediaPipe) to quantify movement toward the midline (MTM) in video of infants taken by their parents in the home. Recent research (Ferrari et al.) has shown a correlation between a type of MTM, foot-to-foot (FF) contact, and Prechtl’s fidgety movements, long considered a hallmark of neuromotor development, and the absence of which is cause for concern. FF and other MTM movements are intuitively easier to identify than fidgety movements. Hence MTM, in conjunction with a readily available video pose estimation model, present a promising opportunity for algorithmic detection of potential delay that is low cost and extensible to families to decrease time to identification and intervention. Authors recruit and consent families to take supine videos of their young infants for classification and measurement of their MTM. Each video is human-labeled according to whether (and which) MTM are present. Subsequently, infant anatomical key point x,y coordinates are generated using the MediaPipe framework and an imaginary midline of the infant’s body calculated. Authors measure the relative frequency of MTM in various quadrants of interest and report the correlation between labeled MTM and distances and areas generated from the MediaPipe coordinates. Correlations were generally statistically significant, but weak. Authors discuss reasons that might explain the results and propose next steps for further investigation.

This work adds to the knowledge base of family-centered technology design and development. There has been a lot of work done using high-precision 3D camera systems to establish the credibility of classifying general movements (GM) algorithmically. So far, this work has had little clinical (therapeutic) utility due to the time, complexity and cost it would take to implement it. There is a great need for lighter weight, more extensible technologies that can be readily utilized in everyday practice (or infants’ homes) with everyday technology. That it is possible and feasible to quantify MTM, as a correlate of GM, from home video using an open-source deep learning model augmented by custom Python code is encouraging. The accessibility of such approaches promises to improve access to care for infants with suspected or unidentified risks for neuromotor developmental delay.

**Specific Areas for Improvement**

In the follow sections I list out what I feel are specific areas where the paper might be improved. They generally follow the organization of the paper itself and my comments in authors’ PDF. I will skip over any comments I made on language clarity since I have a dedicated section on that aspect at the end.

1. There could be greater clarity in describing the relationship that is thought to exist among MTM, pre-voluntary movement, and goal-directed or intentional behaviors. See notes on page 4. I feel like there is some context missing here as well. In GMs, we are looking at non-goal directed movement/behavior. Once the baby becomes capable of intention, the utility of the GM assessment (GMA) wanes. I think this stands in opposition to your statement in the second sentence at line 75. Much of the difficulty in conducting other assessments, such as the Hand Assessment for Infants (HAI) is that measurement is only valid if the infant has a goal driving the measured movement. Hence it is only attempted with older (>3 mos) infants.
2. Lines 72-73. It would be much clearer if you could summarize Ferrari et al.’s findings on specificity and sensitivity. Referencing “3 out of 18” leaves the reader wondering about the other relationships.
3. Focus on 2D. Here’s my comment attached to lines 91-92. If you are going to talk specifically about 2D pose estimation, you pretty much need to acknowledge the large efforts being made in the area of 3D pose estimation. Cameras with depth sensors (ex the Intel RealSense) and two-camera techniques abound. Like marker-based pose estimation, 3D markerless techniques are more costly and difficult to implement than are 2D methods. This absence of discussion seems like a gap. See also the comment at line 98, same basic topic.
4. MediaPipe implementation Details. Lines 108-109 are the first place I begin to feel unsatisfied with the description of the MediaPipe process. Issues of confidence level, visibility (occlusion) are huge and they aren’t foreshadowed here or addressed elsewhere. See also comment at line 193.
6. Rationale for study. I feel your rationale could be expanded somewhat. See comment lines 113-114. The Introduction finishes without a discussion of the need for simple, smartphone camera video analysis to identify possible developmental delay.
7. Harking back to item 4 above, I feel more information is needed on why pose estimation might not have been possible for some videos. See comment line 135.
8. I would like more information on instructions given parents about shooting video? Did you specify a frame rate? It looks like you ended up with 30 fps video at a certain resolution. Did you manipulate the video or instruct parents to set their phone cameras to those parameters? Comment line 141.
9. Human subjects narrative. See comment p 7 attached at line 150. I feel the detail on how participants’ autonomy and privacy were respected is excessive. The reader would be satisfied with a simple statement and the space could be better used I feel.
10. Inopportune breaks across video seconds. See comment lines 177-178. It seems possible that an MTM could be missed if it occurred over frames that split across the 1-second designation.
11. Description of quantification of MTM. See comments lines 178 and 179. It wasn’t clear to me what was meant by “total time interval.” Can you clarify?
12. Rejection of MediaPipe native center-finding strategy, hip midpoint. I feel like users of MediaPipe will be interested in why you didn’t try to leverage the framework’s built-in mid-point “hack.” Yes, it simulates 3D but provides a way to relate all points of interest to a central point. I am not saying your method is not superior, but I would like a sentence or two on why you didn’t try to leverage MediaPipe’s.
13. Table 1 is a bit confusing. The title seems wrong and the percentiles probably require some comment. See my comments at lines 263 and following.
14. Rejection of parent-provided video! See comment line 359. I feel like we need some discussion of this. It seems that one way of getting better results might be reverting to a testing procedure more like the in-lab ones that produce more reliable data but are unworkable. Another way might be modifying the analytic method. Here is where I am particularly wanting to hear about how you valiated the x,y coordinates MediaPipe provided.
15. Figures. Generally they could be clearer, meaning, less fuzzy. Figure 5 is particularly difficult to read unfortunately.
16. Conclusions. I am disappointed in the abandonment of the hugely user-aligned component of your study without a discussion of the tradeoffs. It would be more satisfying to discuss what is being given up for perceived need of greater analytic precision.

**Clarity and Language**

Overall, the use of English is excellent but there are a few expressions that could be more clear and idiomatic. PLOS includes this note in their instructions to reviewers: “PLOS ONE does not copyedit accepted manuscripts, so the language in submitted articles must be clear, correct, and unambiguous. Any typographical or grammatical errors should be corrected at revision, so please note any specific errors here.” Consequently, as I read through the manuscript, I made note of places where I thought expression could be clearer. I recognize that this is a burdensome problem, but it obscures the compelling work you have done. Have you thought of using a large language model (LLM) as an editorial assistant? I copied two sentences that would be unlikely to jeopardize your intellectual property and asked ChatGPT if they could be improved. I think the result is quite impressive. At a minimum, it provides an alternative phrasing for you to consider.

RESPONSE:

Certainly! The passage you provided is technically correct but can be made more readable and concise. Here's a revised version:

"Using specialized techniques like three-dimensional motion capture with wearable sensors to gather quantitative data can lead to high production costs, necessitating a specific setting and expertise [15]. For example, Kanemaru et al. [16] employed a motion analysis system to examine endpoint movement, focusing on individual limbs and interlimb coordination in healthy infants aged 2 to 4 months. They analyzed these indices as precursors to goal-directed movement."

Key changes:

1. Replaced "utilizing" with "using" for simplicity.

2. Streamlined the sentence structure for clarity.

3. Clarified the example provided for better understanding.

4. Used "employed" instead of "conducted a study using" for conciseness.

5. Revised the description of the study for improved flow and readability.

The idea that motion to the midline is pre-voluntary but thought to be the beginnings of goal-directed, intentional movement is important. It suggests that quantification of FF (and perhaps other early movement to the midline) may be predictive of neurodevelopmental disorders beyond CP. If so, the significance of your work is increased. It would be good to specifically and pointedly note this, perhaps in the Discussion or Conclusions.

Lines 91-92. The discussion of 2D markerless pose estimation seems incomplete without a nod to 3D efforts. Like marker-based methods, they have higher overhead and cost, but many would say significantly better accuracy. See comment in text.
