# Supplementary material for: Correlation between pose estimation features regarding movements towards the midline in early infancy
Source: PLoS One. 2024 Feb 28;19(2):e0299758. doi: 10.1371/journal.pone.0299758 (PMC10901309; doi:10.1371/journal.pone.0299758)
Supplement: Attachment — Submitted filename: 000_Response to Reviewers_20240126.docx [file pone.0299758.s004.docx]

**26^th^ January 2024**

**Dr. Prince Jain**

**Academic Editor**

***PLOS ONE***

**Dear Editor,**

**We greatly appreciate the time and effort you and the reviewers dedicated to assessing our paper critically and are pleased to resubmit the revised version of our manuscript to *PLOS ONE*. Your comments, as well as those of the reviewers, were highly insightful and enabled us to greatly improve the quality of our manuscript. The manuscript has been revised following careful consideration of the reviewers’ comments and suggestions.**

**In the following pages, we have provided our point-by-point response to all comments. The revised sections are highlighted in yellow. We hope that you will find our revised manuscript suitable for publication in *PLOS ONE*.**

**The major revisions in the manuscript are as follows:**

- **We have mainly revised the introduction and discussion sections and clarified the methodology, results, and table.**
- **Introduction: We corrected terminology, clarified information, rephrased content, removed certain sentences, and sequentially organized the text.**
- **Methods: We clarified the information and organized the text sequentially within each subtopic.**
- **Result: We have clarified the information, particularly tables and figures.**
- **Discussion: We have considered the related issues with the presented study result and revised almost the whole part.**

**Please let us know if you require any further information.**

**Reviewer #1**

**The summary and annotated pdf file**:

SPECIFIC COMMENTS:

1*. “There could be greater clarity in describing the relationship that is thought to exist among MTM, pre-voluntary movement, and goal-directed or intentional behaviors. See notes on page 4. I feel like there is some context missing here as well. In GMs, we are looking at non-goal directed movement/behavior. Once the baby becomes capable of intention, the utility of the GM assessment (GMA) wanes. I think this stands in opposition to your statement in the second sentence at line 75. Much of the difficulty in conducting other assessments, such as the Hand Assessment for Infants (HAI) is that measurement is only valid if the infant has a goal driving the measured movement. Hence it is only attempted with older (>3 mos) infants”*

**In accordance with your comment, we have revised the manuscript as follows:**

**Page 4, lines 76-81:**

The occurrence of MTM in early infancy may indicate movement abilities before reaching goal-directed movements in the future. The frequency of MTM occurrences may be associated with sensory-motor exposure. A lower frequency may signify diminished motor-sensory input, while a higher frequency could imply limited movement patterns due to increased repetition and monotony [15].

(References)

15.	Sermpon N, Gima H. Relationship between fidgety movement and frequency of movement toward midline: An observational study. Early Hum Dev. 2023;177–178: 105718. doi:[10.1016/j.earlhumdev.2023.105718](https://doi.org/10.1016/j.earlhumdev.2023.105718)

2. *“Line 72-73. It would be much clearer if you could summarize Ferrari et al.’s findings on specificity and sensitivity. Referencing “3 out of 18” leaves the reader wondering about the other relationships.”*

**In order to focus on typical infants in this study, we have moved references to the Ferrari et al. study from the Introduction to the Discussion and clarified their specificity and sensitivity.**

**Page 16, lines 372-383:**

Ferrari et al. [35] studied the use of FF movements for supporting FMs by assessing both FMs and FF occurrence in infants with hypoxic-ischemic encephalopathy, describing the sensitivity and specificity for CP prediction individually and when pooling the two factors together. The result showed the sensitivity and specificity of both patterns to predict CP was 100% and 73%, respectively. This suggests that FF could serve as a supportive tool, emphasizing the need for further exploration of various FF movements. When considering FF subtypes, such as plant-to-plant contact, plant-to-tibial margin of the contralateral foot, plant-to-dorsal part of the contralateral foot, feet crossing, and pedipulation, more specific information about infant development can be obtained [35]. Meanwhile, the FL components exhibit higher development, involving lifting the leg and crossing over to touch the opposite side. The repetition of this movement pattern could lead to the rolling component [36].

(References)

35.	Ferrari F, Bedetti L, Bertoncelli N, Roversi MF, Della Casa E, Guidotti I, et al. Foot-to-foot contact among initial goal-directed movements supports the prognostic value of fidgety movements in HIE-cooled infants. Front Pediatr. 2022;9. doi:[10.3389/fped.2021.731021](https://doi.org/10.3389/fped.2021.731021)

36.	Kobayashi Y, Watanabe H, Taga G. Movement patterns of limb coordination in infant rolling. Exp Brain Res. 2016;234: 3433–3445. doi:10.1007/s00221-016-4741-2

3. *“Focus on 2D. Here’s my comment attached to lines 91-92. If you are going to talk specifically about 2D pose estimation, you pretty much need to acknowledge the large efforts being made in the area of 3D pose estimation. Cameras with depth sensors (ex the Intel RealSense) and two-camera techniques abound. Like marker-based pose estimation, 3D markerless techniques are more costly and difficult to implement than are 2D methods. This absence of discussion seems like a gap. See also the comment at line 98, same basic topic”*

**In accordance with your comment, we have revised the manuscript as follows:**

**Page 5, lines 100-112:**

Recently, human pose estimation has gained widespread use and found applications in various fields, such as sports, home care, work monitoring, and surveillance [23,24]. The advantages of two dimensional (2D) and 3D human pose estimation depend on the application objective. 2D human pose estimation is an alternative computer vision technique used to estimate the pose of a person in a 2D image or video [25,26]. This is a real-time detection method based on the x and y coordinates of human body joints from RGB (red, green, and blue) images [27]. The study of marker-free infant movement has shown advantages in which infants perform actions naturally with minimal cost, less time consumption, and measurable quantitative data [28,29]. 3D pose estimation can be achieved using a depth camera (e.g. Microsoft Kinect, Intel RealSense, etc.) or Motion capture system [24,30]. Similar to marker-based systems, employing markerless 3D techniques is more challenging to implement compared with 2D methods. 3D pose estimation may require proper training, expertise, and high computational complexity for accurate analysis.

(References)

23.	Kim J-W, Choi J-Y, Ha E-J, Choi J-H. Human pose estimation using MediaPipe pose and optimization method based on a humanoid model. Appl Sci. 2023;13: 2700. doi:[10.3390/app13042700](https://doi.org/10.3390/app13042700)

24.	Ben Gamra M, Akhloufi MA. A review of deep learning techniques for 2D and 3D human pose estimation. Image Vis Comput. 2021;114: 104282. doi: [10.1016/j.imavis.2021.104282](https://doi.org/10.1016/j.imavis.2021.104282)

25.	Stenum J, Cherry-Allen KM, Pyles CO, Reetzke RD, Vignos MF, Roemmich RT. Applications of pose estimation in human health and performance across the lifespan. Sensors (Basel). 2021;21: 7315. doi:[10.3390/s21217315](https://doi.org/10.3390/s21217315)

26.	Sermpon N, Gima H. A systematic review of the measurement of infant posture and movement using image or video data analysis | Archives of allied health sciences. Arch AHS. 2022;34: 1–15.

27.	Cao Z, Hidalgo G, Simon T, Wei S-E, Sheikh Y. OpenPose: Realtime multi-person 2D pose estimation using part affinity fields. IEEE Trans Pattern Anal Mach Intell. 2021;43: 172–186. doi:[10.1109/TPAMI.2019.2929257](https://doi.org/10.1109/tpami.2019.2929257)

28.	Hesse N, Sebastian Schroeder A, Müller-Felber W, Bodensteiner C, Arens M, G. Hofmann U. Markerless motion analysis for early detection of infantile movement disorders. In: Eskola H, Väisänen O, Viik J, Hyttinen J, editors. EMBEC & NBC 2017. Singapore: Springer; 2018. pp. 197–200. doi:10.1007/978-981-10-5122-7_50

29.	Reich S, Zhang D, Kulvicius T, Bölte S, Nielsen-Saines K, Pokorny FB, et al. Novel AI driven approach to classify infant motor functions. Sci Rep. 2021;11: 1–13. doi:[10.1038/s41598-021-89347-5](https://doi.org/10.1038/s41598-021-89347-5)

30.	Marcroft C, Khan A, Embleton ND, Trenell M, Plötz T. Movement recognition technology as a method of assessing spontaneous general movements in high risk infants. Front Neurol. 2015;5: 284. doi:[10.3389/fneur.2014.00284](https://doi.org/10.3389%2Ffneur.2014.00284)

4. *“MediaPipe implementation Details. Lines 108-109 are the first place I begin to feel unsatisfied with the description of the MediaPipe process. Issues of confidence level, visibility (occlusion) are huge and they aren’t foreshadowed here or addressed elsewhere. See also comment at line 193.”*

**In light of your comment, we have added information about using Google MediaPipe for pose estimation as follows:**

**Page 5, lines 113-124, and Page 6, lines 125-129:**

There are few MTM studies with both quality and quantity. In the present study, we observed and applied an open source, pose estimation model (google MediaPipe) to quantify MTM in videos of infants taken on their parents’ mobile phones at home. MediaPipe pose is a machine-learning solution capable of tracking key body parts in images or videos, and generating 33 pose landmarks presented in either normalized image coordinates or world coordinates [31]. In this study, we simply employed 2D pose estimation and computed distance and area using distal limb landmark coordinates. When infants show MTM in videos, an observable reduction in the distance between the distal part of limbs and the midbody imaginary line is seen. Furthermore, when measuring the spatial area defined by the endpoint of the extremity and midpoint of the body, MTM movements may reduce the enclosed area when compared with instances where the limbs are positioned farther from the midpoint. This study aimed to identify the correlation between features from pose estimation data (i.e. distance and area), and infant movement observations regarding MTM, in infants at an early age (8–16 weeks PTA). We explored the observed MTM values, and the distances calculated through open source 2D pose estimation coordinates, to determine whether a relationship exists or not. Moreover, we identified a disparity in the area between the videos that displayed MTM and those that did not.

(Reference)

31.	Pose landmark detection guide | MediaPipe. In: Google for Developers [Internet]. [cited 6 Sep 2023]. Available: https://developers.google.com/mediapipe/solutions/vision/pose_landmarker

**Regarding the issues related to visibility, we have added the following descriptions to the methods and limitations of the study:**

**Page 7, lines 170-173:**

Videos were excluded due to visibility issues, such as infants energetically moving their limbs beyond the camera frame, leading to the program's inability to detect corresponding landmark coordinates. Furthermore, if the program failed to execute the code in its entirety, resulting in an incomplete analysis, the video was excluded.

**Page 18, lines 433-434, and Page 19, lines 435-451:**

This study had some limitations. First, video recording was done by a parent or guardian; hence, it was common to have some interferences that could not be avoided due to their presence when approaching infants of this age. Infants might react and need a response from the person who was taking a video. Some of them stared at the mobile phone and did not show any movement. Second, despite efforts to maintain a perpendicular position for handheld smartphones during recording, slight movements or changes in camera angles owing to the camera-holding hand were possible. However, since the features calculated in this study are angular information obtained by connecting markers with lines and distance information corrected by body length, we believe that their influence was minimized. Hand-holding a camera may have been a convenient and realistic method for parents or caregivers. However, if they had to set up a camera tripod or control the camera similar to a laboratory setting, this may have become burdensome. Improving the capacity to adapt to factors such as camera angle and occlusion is essential for optimizing the functionality of a lightweight, parent-controlled system in identifying neuromotor delays in infants. This enhanced ability ensures more robust performance, enabling accurate assessments even in challenging conditions. Third, the sample size appears to be limited, and as a result, the presented findings may not accurately represent all infants. Further study is required with a larger number of infants and an extended duration of follow-up to thoroughly assess the effectiveness of the 2D pose estimation method in extracting values related to MTM.

5. *“Rationale for study. I feel your rationale could be expanded somewhat. See comment lines 113-114. The Introduction finishes without a discussion of the need for simple, smartphone camera video analysis to identify possible developmental delay.”*

**In light of your comment, we have added information about the study rationale as follows:**

**Page 5, lines 113-124, and Page 6, lines 125-138:**

There are few MTM studies with both quality and quantity. In the present study, we observed and applied an open source, pose estimation model (google MediaPipe) to quantify MTM in videos of infants taken on their parents’ mobile phones at home. MediaPipe pose is a machine-learning solution capable of tracking key body parts in images or videos, and generating 33 pose landmarks presented in either normalized image coordinates or world coordinates [31]. In this study, we simply employed 2D pose estimation and computed distance and area using distal limb landmark coordinates. When infants show MTM in videos, an observable reduction in the distance between the distal part of limbs and the midbody imaginary line is seen. Furthermore, when measuring the spatial area defined by the endpoint of the extremity and midpoint of the body, MTM movements may reduce the enclosed area when compared with instances where the limbs are positioned farther from the midpoint. This study aimed to identify the correlation between features from pose estimation data (i.e. distance and area), and infant movement observations regarding MTM, in infants at an early age (8–16 weeks PTA). We explored the observed MTM values, and the distances calculated through open source 2D pose estimation coordinates, to determine whether a relationship exists or not. Moreover, we identified a disparity in the area between the videos that displayed MTM and those that did not. The hypotheses were that the occurrence percentage and rate per minute of MTM would be associated with the distance between distal joints and the midbody imaginary line. Moreover, the area values in the videos showing lower-part MTM were lower than those in videos without showing MTM, as well as in videos showing upper MTM. This study may be practical and useful to family-centre services for following up infant development with straightforward movement patterns. Utilizing 2D pose estimation can offer a more lightweight and adaptable technology that can be easily applied in everyday practice, especially in low-resource settings, using contemporary technology. The accessibility of such approaches promises to improve access to care for infants with suspected or unidentified risks for neuromotor developmental delay.

(References)

31.	Pose landmark detection guide | MediaPipe. In: Google for Developers [Internet]. [cited 6 Sep 2023]. Available: https://developers.google.com/mediapipe/solutions/vision/pose_landmarker

6. *“Harking back to item 4 above, I feel more information is needed on why pose estimation might not have been possible for some videos. See comment line 135.”*

**In accordance with your comment, we have revised the manuscript as follows:**

**Page 7, lines 169 – 173:**

The initial 5 s of the video were cut off and subsequently maintained for 2 mins thereafter. Videos were excluded due to visibility issues, such as infants energetically moving their limbs beyond the camera frame, leading to the program's inability to detect corresponding landmark coordinates. Furthermore, if the program failed to execute the code in its entirety, resulting in an incomplete analysis, the video was excluded.

7. *“I would like more information on instructions given parents about shooting video? Did you specify a frame rate? It looks like you ended up with 30 fps video at a certain resolution. Did you manipulate the video or instruct parents to set their phone cameras to those parameters? Comment line 141.”*

**Parents or guardians were instructed to use general camera mode which is normally set at 30 frames per second as some smartphones have the function of 60 frames per second.**

**Page 7, lines 156-162:**

Parents and guardians were instructed to record videos of the infants using the video function of their smartphones, setting the camera to a common configuration of 30 frames per s. First, they were asked to dress infants in a nappy or a bodysuit and place them on a firm mat in the supine position. Next, they had to hold the mobile camera and position it approximately 1 m above the infant, ensuring a top view covering all the infant's limbs. They were instructed to record a video for 2–3 min and avoid interacting with the infant while holding the mobile phone. No specific instructions were given to the guardians regarding when to start recording.

8. *“Human subjects narrative. See comment p 7 attached at line 150. I feel the detail on how participants’ autonomy and privacy were respected is excessive. The reader would be satisfied with a simple statement and the space could be better used I feel.”*

**In accordance with your comment, we have revised the text as follows:**

**Page 7, lines 163-165:**

Afterwards, the video file was securely transferred to the researcher through cloud storage using multiple security settings on Dropbox. Participants' autonomy and privacy were respected, with careful measures implemented to safeguard the confidentiality of infant data.

9. *“Inopportune breaks across video seconds. See comment lines 177-178. It seems possible that an MTM could be missed if it occurred over frames that split across the 1-second designation.”*

**In accordance with your comment, we have clarified the observation time as follows:**

**Page 8, lines 192-195:**

All videos were examined for frame count, with a total of 3600 frames. The 10 MTM 10 items were observed, with each item requiring a presence of 30 frames continuously to be counted as one second. Wherein an MTM item fell below 30 frames, it was excluded from the count.

10. *“Description of quantification of MTM. See comments lines 178 and 179. It wasn’t clear to me what was meant by “total time interval.” Can you clarify?”*

**In accordance with your comment, we have revised this section as follows:**

**Page 8, lines 192-199, and Page 9, lines 200-208:**

All videos were examined for frame count, with a total of 3600 frames. The 10 MTM items were observed, with each item requiring a presence of 30 frames continuously to be counted as one second. When an MTM item fell below 30 frames, it was excluded from the count. The occurrence times of each MTM were summed up, and the total occurrence time for the 10 MTMs was calculated. The MTM occurrence percentage for each video was determined using the following equation, where 120 represents the video duration in seconds:

MTM occurrence percentage = (Total MTM occurrence time (s))/(120 (s))×100	(1)

MTM involves contact between limbs or limbs and the body, suggesting a potential connection to sensory-motor input. We aimed to assess the frequency of these occurrences and understand the patterns exhibited with rate per min. The MTM occurrence rate per min was calculated and divided into three groups: total MTM occurrence rate per min, lower-limb MTM occurrence rate per min ( FF, FL, and pedipulation), and upper-limb MTM occurrence rate per min (HH, HF, HM, HT, HL, manipulation, and fiddling) [15].

MTM occurrence rate per minute = (MTM occurrence time (s): Total, Lower, Upper MTM)/(120 (s))×60 (2)

11. *“Rejection of MediaPipe native center-finding strategy, hip midpoint. I feel like users of MediaPipe will be interested in why you didn’t try to leverage the framework’s built-in mid-point “hack.” Yes, it simulates 3D but provides a way to relate all points of interest to a central point. I am not saying your method is not superior, but I would like a sentence or two on why you didn’t try to leverage MediaPipe’s.”*

**This sentence refers to finding midbody coordinates using area computation. We did apply MediaPipe functions and simply computed them using all landmark coordinates. We have revised this section to further clarify this point as follows:**

**Page 9, lines 211-223, and Page 10, lines 224-237 (Procedure of pose estimation and distance calculation):**

In this study, Python (version 3.9.12) was applied and we processed 2D pose estimation using the internal MediaPipe function [31] to extract 33 body landmark coordinates (x, y) from each 2-min video. We set the minimum confidence score as 0.5 for pose detection. The landmarks were computed, utilising normalised screen coordinates obtained from the x and y coordinates. Consequently, these points yielded indices with dimensionless values. After running the code, we obtained 118, 800 coordinates in comma-separated value (csv) files. We then checked each file, eliminating those with incomplete data, and excluding the corresponding videos. The selection of wrist and ankle coordinates (landmarks 15 and 16 and 27 and 28, respectively) were used to calculate the distance between the limbs and midbody imaginary line. First, we determined the midpoint of the shoulder and hip, landmarks 11 and 12 and 23 and 24, respectively, based on the midpoint formula:

(x_mSh,y_mSh ) =((x_11+ x_12)/2,(y_11+ y_12)/2)	(3)

(x_mH,y_mH ) =((x_23+ x_24)/2,(y_23+ y_24)/2)	(4)

where (x_mSh,y_mSh ) represented the midpoint coordinate of the shoulder and (x_mH,y_mH ) represented the midpoint coordinate of the hip. Then, to find the midbody imaginary line, we applied both midpoint (x, y) values to the line equation:

y = mx + c	(5)

substituting the y value of limb landmark coordinates (15 and 16 for the wrists, 27 and 28 for the ankles) to obtain the x value at the midbody imaginary line, followed by subtracting x of the landmarks to obtain the distance (Fig 1). All distance values were normalised to the average of the left and right body lengths (Euclidean distances of landmarks 11–23, 12–24). After obtaining the distance values from all the frames, we calculated the average distance of each of the four landmarks from the midline. Therefore, each video (120 s) had a single average number representing the distance from the midline of the four selected landmarks. Hence, the distance of landmark (Dlm) 15 represents the distance between landmark 15 and the midbody imaginary line, Dlm16 represents the distance between landmark 16 and the midbody imaginary line, and so on for Dlm27 and Dlm28.

(Reference)

31.	Pose landmark detection guide | MediaPipe. In: Google for Developers [Internet]. [cited 6 Sep 2023]. Available: <https://developers.google.com/mediapipe/solutions/vision/pose_landmarker>

**Page 10, lines 243-248, and Page 11, lines 249-257 (Procedure of pose estimation and finding body areas):**

In this study, our focus encompassed the entire body, including the upper and lower limb regions (Fig 2). Initially, we utilized the internal MediaPipe function in Python (version 3.9.12) for 2D pose estimation, resulting in 33 landmarks. Subsequently, we selected wrist and ankle landmarks (15, 16, 27, and 28) to compute the quadrilateral area representing the whole area (Fig 2A). The centre of body coordinates were calculated using all landmark coordinates. Following this, we calculated the triangular area for the upper part using landmarks 15 and 16 and the centre of the body coordinate, as well as the lower part using landmarks 27 and 28 and the centre of the body coordinates (Fig 2B). All area values were normalized based on the average of the left and right body lengths. From each video, we computed the average area values for the whole area, upper part, and lower part.

**Fig 2.** **Pose landmark** [31] **and calculated area.** This figure shows calculated area using limbs pose landmarks (15, 16, 27, and 28) and centre of the body coordinate which are listed as: (**A**) presenting the whole area; (**B**) presenting ➊ = the upper part area, and ➋ = the lower part area.

12. *“Table 1 is a bit confusing. The title seems wrong and the percentiles probably require some comment. See my comments at lines 263 and following.”*

**In accordance with your comment, we have revised this table as follows:**

**Page 13, lines 296-300:**

**Table 1.** **Anthropometric data at birth according to the 2006 WHO Child Growth Standards: 0-6 months (weight and length) and 0-13 weeks (head circumference) [32] (N = 20)**

|  | **Gestational age**  **(weeks + days)** | **Weight (g)**  (range of percentile) | **Length (cm)**  (range of percentile) | **Head circumference (cm)**  (range of percentile) |
| --- | --- | --- | --- | --- |
| **Median** | 38 + 4 | 3150  (P15-P50) | 49  (P15-P50) | 34  (P50-P85) |
| **Min** | 37 + 0 | 2550  (P3-P15) | 47  (P3-P15) | 31  (<P3) |
| **Max** | 40 + 1 | 3900  (P85-P97) | 55  (>P97) | 36.5  (>P97) |

g = grams, cm = centimetres, P3 = 3^rd^ percentile, P15 = 15^th^ percentile, P50 = 50^th^ percentile, P85 = 85^th^ percentile, P97 = 97^th^ percentile

(Reference)

32.	The WHO Child Growth Standards. [cited 5 Sep 2023]. Available: https://www.who.int/tools/child-growth-standards

13. *“Rejection of parent-provided video! See comment line 359. I feel like we need some discussion of this. It seems that one way of getting better results might be reverting to a testing procedure more like the in-lab ones that produce more reliable data but are unworkable. Another way might be modifying the analytic method. Here is where I am particularly wanting to hear about how you valiated the x,y coordinates MediaPipe provided?”*

**In accordance with your comment, we have added the following limitations to the study:**

**Page 18, lines 433-434, and Page 19, lines 435-451:**

This study had some limitations. First, video recording was done by a parent or guardian; hence, it was common to have some interferences that could not be avoided due to their presence when approaching infants of this age. Infants might react and need a response from the person who was taking a video. Some of them stared at the mobile phone and did not show any movement. Second, despite efforts to maintain a perpendicular position for handheld smartphones during recording, slight movements or changes in camera angles owing to the camera-holding hand were possible. However, since the features calculated in this study are angular information obtained by connecting markers with lines and distance information corrected by body length, we believe that their influence was minimized. Hand-holding a camera may have been a convenient and realistic method for parents or caregivers. However, if they had to set up a camera tripod or control the camera similar to a laboratory setting, this may have become burdensome. Improving the capacity to adapt to factors such as camera angle and occlusion is essential for optimizing the functionality of a lightweight, parent-controlled system in identifying neuromotor delays in infants. This enhanced ability ensures more robust performance, enabling accurate assessments even in challenging conditions. Third, the sample size appears to be limited, and as a result, the presented findings may not accurately represent all infants. Further study is required with a larger number of infants and an extended duration of follow-up to thoroughly assess the effectiveness of the 2D pose estimation method in extracting values related to MTM.

14. *“Figures. Generally they could be clearer, meaning, less fuzzy. Figure 5 is particularly difficult to read unfortunately.”*

**In light of your comment, we have revised the figure legend of Figure 5 to improve readability as follows:**

**Page 15, lines 336-349:**

**Fig 5.** **The difference between groups of videos that showed MTM and those that did not.**

(A) The Lower MTM group represents infants showing foot-to-foot contact, foot-to-leg contact, and pedipulation. The comparison of whole area and upper- and lower-part areas between the groups of videos that showed MTM and those that did not are presented.

(B) The Upper MTM group represents infants showing hand-to-hand, hand-to-face, hand-to-mouth, hand-to-trunk, hand-to-leg, manipulation, and fiddling. The comparison of whole area and upper- and lower-part areas between the groups of videos that showed MTM and those that did not are presented.

(C) The Total MTM group represents infants showing all 10 items: foot-to-foot contact, foot-to-leg contact, hand-to-hand, hand-to-face, hand-to-mouth, hand-to-trunk, hand-to-leg, pedipulation, manipulation, and fiddling. The comparison of whole area and upper- and lower-part areas between groups of videos that showed MTM and those that did not are presented.

MTM = movement toward midline, * p-value < 0.05. The sign of ■ represents the group of videos showing MTM and  represents the group of videos not showing MTM.

15. *“Conclusions. I am disappointed in the abandonment of the hugely user-aligned component of your study without a discussion of the tradeoffs. It would be more satisfying to discuss what is being given up for perceived need of greater analytic precision.”*

**In accordance with your comment, we have revised this section follows**.

**Page 19 line 454-459, and Page 20, lines 460-469:**

Assessing movement in infants at an early age plays an important role in detecting motor disorders. The emergence of MTM during spontaneous movement may support intentional movement development. The novel approach of markerless movement analysis has the potential to enhance the developmental follow-up system. In this study, infant video recordings were captured by parents or guardians. Open source 2D pose estimation features (coordinates, distance, and area) were utilised to provide support in identifying the presence of MTM in infants. We observed a significant correlation between MTM observation values and distances calculated from limb landmarks to the midbody imaginary line. Notably, both the whole area and the lower part area exhibited significantly lower values in the videos showing MTM, especially in the Lower MTM group. This method offers advantages for natural infant movement with non-invasive techniques, lightweight computational analysis, and regular follow-up, particularly in isolated areas or difficult situations. However, the development of 2D landmarks into 3D pose estimation is challenging due to the complexity of MTM movements characterized by antigravity patterns. In future studies, a larger sample size and inclusion of high-risk infants are essential to assess MTM patterns and differentiate between pathological and typical MTM movements.

**Reviewer #2**

*“The study uses of 2D pose estimation for analyzing infant movements. However, significant revisions are needed to address the concerns raised, particularly regarding methodology, data analysis, and the discussion of results.”*

SPECIFIC COMMENTS:

1. *“The Introduction section should more effectively set the stage for the research, outlining the current state of knowledge, identifying gaps, and explicitly stating the research question or hypothesis*.*”*

**In accordance with your comment, we have revised the Introduction as follows.**

**Pages 2-6, lines 47-138:**

Infant posture and spontaneous movements hold significance in early development [1]. General movements (GMs) [2], that is whole-body spontaneous movements, have been studied and a method has been developed for detecting neuromotor impairment in the first 5 months post-term age (PTA). The Prechtl general movements assessment (GMA) is an observational method clinically used worldwide. This tool was approved as a validated indicator [3,4], particularly regarding fidgety movements (FMs, small-moderate speed movements, and varying accelerations in all directions across the neck, trunk, and limbs), for identifying the occurrence of neurodevelopment problems (e.g. cerebral palsy [CP]) or movement disorders [5–8]. Examining additional postural and movement factors could contribute to supporting and reinforcing the early diagnosis of CP [9]. The movement optimality scale [2,10] was established and revised based on the GMA, with scores ranging from 5 to 28. A decrease in the score may indicate a higher risk of motor or cognitive impairment [11,12]. Although this assessment result is predominantly based on the presence of FMs, it also considers various concurrent postural and movement patterns observed alongside GMs, which may affect the overall score. These encompass the alignment of the head, body, and limbs, as well as asymmetric tonic neck posture (ATNP) and limb movements, such as movement toward the midline (MTM) (foot-to-foot [FF], fiddling, hand-to-hand [HH], and hand-to-mouth [HM] contact) [2,12,13]. A study evaluating concurrent motor and postural patterns with GMs for the early diagnosis of CP found that MTM in both the upper and lower limbs could be considered at fidgety age, following monotonous and stereotyped limb movements [9].

MTM is characterised by limb activities moving against gravity and close to the imaginary midline which symmetrically divides the body into left and right sides. In early infancy, this motor pattern was considered initially developmental toward intentional movement behaviour and oriented toward specific objectives. Lucaccioni et al. [14] expanded the understanding of the typical and age-adequate motor repertoire regarding MTM of the four limbs by observing and identifying the developmental progression in healthy, full-term infants from birth until 22 to 23 weeks PTA. Infants were observed to have 10 MTM in writhing and fidgety movement periods, including: FF, foot-to-leg (FL), HH, hand-to-face (HF), HM, hand-to-trunk (HT), hand-to-leg (HL), manipulation, pedipulation, and fiddling. Progressive increases in frequencies were notably observed, particularly during the age of FMs. The occurrence of MTM in early infancy may indicate movement abilities before reaching goal-directed movements in the future. The frequency of MTM occurrences may be associated with sensory-motor exposure. A lower frequency may signify diminished motor-sensory input, while a higher frequency could imply limited movement patterns due to increased repetition and monotony [15].

Movement analysis exhibits a wide range of diversity, and its level of accessibility depends on numerous elements, encompassing the assessor, the client, available resources, locations, technological advancements, budgetary considerations, and other relevant factors. Previous studies [16–18] demonstrated various movement assessment methods, including visual observation, wearable sensor technology systems, and markerless systems for infant movement monitoring. Using specialized techniques, like three-dimensional (3D) motion capture with wearable sensors, to gather quantitative data can lead to high production costs, necessitating a specific setting and expertise [19]. For example, Kanemaru et al. [20] employed a motion analysis system to examine endpoint movement, focusing on individual limbs and interlimb coordination in healthy infants aged between 2 and 4 months. They analysed these indices as precursors to goal-directed movement. Miyagishima et al. [21] performed a comparison between preterm (corrected age: 1–3 months) and term infants, utilising sensors and a 3D motion capture system, to analyse the characteristics of antigravity spontaneous movements, which encompassed measurements of distance and height for both hands and feet. Marchi et al. [22] examined the predictive capability of quantitative computerised kinematic indices in a group of 12 low-risk term infants using a motion analysis system with six markers. The analysis was done by interpreting indexes of coordination, distance, and global movement quality based on the infants’ motions toward the midline.

Recently, human pose estimation has gained widespread use and found applications in various fields, such as sports, home care, work monitoring, and surveillance [23,24]. The advantages of two-dimensional (2D) and 3D human pose estimation depend on the application objective. 2D human pose estimation is an alternative computer vision technique used to estimate the pose of a person in a 2D image or video [25,26]. This is a real-time detection method based on the x and y coordinates of human body joints from RGB (red, green, and blue) images [27]. The study of marker-free infant movement has shown advantages in which infants perform actions naturally with minimal cost, less time consumption, and measurable quantitative data [28,29]. 3D pose estimation can be achieved using a depth camera (e.g. Microsoft Kinect, Intel RealSense, etc.) or Motion capture system [24,30]. Similar to marker-based systems, employing markerless 3D techniques is more challenging to implement compared with 2D methods. 3D pose estimation may require proper training, expertise, and high computational complexity for accurate analysis.

There are few MTM studies with both quality and quantity. In the present study, we observed and applied an open source, pose estimation model (google MediaPipe) to quantify MTM in videos of infants taken on their parents’ mobile phones at home. MediaPipe pose is a machine-learning solution capable of tracking key body parts in images or videos, and generating 33 pose landmarks presented in either normalized image coordinates or world coordinates [31]. In this study, we simply employed 2D pose estimation and computed distance and area using distal limb landmark coordinates. When infants show MTM in videos, an observable reduction in the distance between the distal part of limbs and the midbody imaginary line is seen. Furthermore, when measuring the spatial area defined by the endpoint of the extremity and midpoint of the body, MTM movements may reduce the enclosed area when compared with instances where the limbs are positioned farther from the midpoint. This study aimed to identify the correlation between features from pose estimation data (i.e. distance and area), and infant movement observations regarding MTM, in infants at an early age (8–16 weeks PTA). We explored the observed MTM values, and the distances calculated through open source 2D pose estimation coordinates, to determine whether a relationship exists or not. Moreover, we identified a disparity in the area between the videos that displayed MTM and those that did not. The hypotheses were that the occurrence percentage and rate per minute of MTM would be associated with the distance between distal joints and the midbody imaginary line. Moreover, the area values in the videos showing lower-part MTM were lower than those in videos without showing MTM, as well as in videos showing upper MTM. This study may be practical and useful to family-centre services for following up infant development with straightforward movement patterns. Utilizing 2D pose estimation can offer a more lightweight and adaptable technology that can be easily applied in everyday practice, especially in low-resource settings, using contemporary technology. The accessibility of such approaches promises to improve access to care for infants with suspected or unidentified risks for neuromotor developmental delay.

(References)

1.	Cioni G, Ferrari F, Prechtl HFR. Posture and spontaneous motility in fullterm infants. Early Hum Dev. 1989;18: 247–262. doi:[10.1016/0378-3782(89)90020-0](https://doi.org/10.1016/0378-3782(89)90020-0)

2.	Einspieler C, Prechtl HFR, Bos A, Ferrari F, Cioni G. Prechtl’s method on the qualitative assessment of general movements in preterm, term and young infants. Clin Dev Med. 2004;167: 1–91.

3.	Bosanquet M, Copeland L, Ware R, Boyd R. A systematic review of tests to predict cerebral palsy in young children. Developmental Medicine & Child Neurology. 2013;55: 418–426. doi:[10.1111/dmcn.12140](https://doi.org/10.1111/dmcn.12140)

4.	Kwong AKL, Fitzgerald TL, Doyle LW, Cheong JLY, Spittle AJ. Predictive validity of spontaneous early infant movement for later cerebral palsy: a systematic review. Dev Med Child Neurol. 2018;60: 480–489. doi:[10.1111/dmcn.13697](https://doi.org/10.1111/dmcn.13697)

5.	Cioni G, Prechtl HF. Preterm and early postterm motor behaviour in low-risk premature infants. Early Hum Dev. 1990;23: 159–191. doi:[10.1016/0378-3782(90)90012-8](https://doi.org/10.1016/0378-3782(90)90012-8)

6.	Einspieler C, Prechtl HFR. Prechtl’s assessment of general movements: A diagnostic tool for the functional assessment of the young nervous system. Ment Retard Dev Disabil Res Rev. 2005;11: 61–67. doi:[10.1002/mrdd.20051](https://doi.org/10.1002/mrdd.20051)

7.	Novak I, Morgan C, Adde L, Blackman J, Boyd RN, Brunstrom-Hernandez J, et al. Early, accurate diagnosis and early intervention in cerebral palsy: Advances in diagnosis and treatment. JAMA Pediatr. 2017;171: 897–907. doi:[10.1001/jamapediatrics.2017.1689](https://doi.org/10.1001/jamapediatrics.2017.1689)

8.	Einspieler C, Marschik PB, Bos AF, Ferrari F, Cioni G, Prechtl HF. Early markers for cerebral palsy: Insights from the assessment of general movements. Future Neurol. 2012;7: 709–717. doi:[10.2217/fnl.12.60](https://doi.org/10.2217/fnl.12.60)

9.	Ferrari F, Plessi C, Lucaccioni L, Bertoncelli N, Bedetti L, Ori L, et al. Motor and postural patterns concomitant with general movements are associated with cerebral palsy at term and fidgety age in preterm infants. J Clin Med. 2019;8: 1189. doi: [doi.org/10.3390/jcm8081189](https://doi.org/10.3390/jcm8081189)

10.	Einspieler C, Bos AF, Krieber-Tomantschger M, Alvarado E, Barbosa VM, Bertoncelli N, et al. Cerebral palsy: Early markers of clinical phenotype and functional outcome. J Clin Med. 2019;8: 1616. doi:[10.3390/jcm8101616](https://doi.org/10.3390/jcm8101616)

11.	Peyton C, Millman R, Rodriguez S, Boswell L, Naber M, Spittle A, et al. Motor optimality scores are significantly lower in a population of high-risk infants than in infants born moderate-late preterm. Early Hum Dev. 2022;174: 105684. doi:[10.1016/j.earlhumdev.2022.105684](https://doi.org/10.1016/j.earlhumdev.2022.105684)

12.	Kwong AK-L, Boyd RN, Chatfield MD, Ware RS, Colditz PB, George JM. Early motor repertoire of very preterm infants and relationships with 2-year neurodevelopment. J Clin Med. 2022;11: 1833. doi:[10.3390/jcm11071833](https://doi.org/10.3390/jcm11071833)

13.	Örtqvist M, Einspieler C, Marschik PB, Ådén U. Movements and posture in infants born extremely preterm in comparison to term-born controls. Early Hum Dev. 2021;154: 105304. doi:[10.1016/j.earlhumdev.2020.105304](https://doi-org.ejournal.mahidol.ac.th/10.1016/j.earlhumdev.2020.105304)

14.	Lucaccioni L, Bertoncelli N, Comini M, Martignoni L, Coscia A, Lugli L, et al. The ontogeny of limbs movements towards midline in healthy infants born at term. Early Hum Dev. 2021;155: 105324. doi:[10.1016/j.earlhumdev.2021.105324](https://doi.org/10.1016/j.earlhumdev.2021.105324)

15.	Sermpon N, Gima H. Relationship between fidgety movement and frequency of movement toward midline: An observational study. Early Hum Dev. 2023;177–178: 105718. doi:[10.1016/j.earlhumdev.2023.105718](https://doi.org/10.1016/j.earlhumdev.2023.105718)

16.	Silva N, Zhang D, Kulvicius T, Gail A, Barreiros C, Lindstaedt S, et al. The future of general movement assessment: The role of computer vision and machine learning – A scoping review. Res Dev Disabil. 2021;110: 103854. doi:[10.1016/j.ridd.2021.103854](https://doi.org/10.1016/j.ridd.2021.103854)

17.	Chen H, Xue M, Mei Z, Bambang Oetomo S, Chen W. A review of wearable sensor systems for monitoring body movements of neonates. Sensors (Basel). 2016;16: 2134. doi:[10.3390/s16122134](https://doi.org/10.3390/s16122134)

18.	Irshad MT, Nisar MA, Gouverneur P, Rapp M, Grzegorzek M. AI approaches towards Prechtl’s assessment of general movements: A systematic literature review. Sensors (Basel). 2020;20. doi:[10.3390/s20185321](https://doi.org/10.3390/s20185321)

19.	Stenum J, Rossi C, Roemmich RT. Two-dimensional video-based analysis of human gait using pose estimation. PLOS Comp Biol. 2021;17: e1008935. doi:[10.1371/journal.pcbi.1008935](https://doi.org/10.1371/journal.pcbi.1008935)

20.	Kanemaru N, Watanabe H, Taga G. Increasing selectivity of interlimb coordination during spontaneous movements in 2- to 4-month-old infants. Exp Brain Res. 2012;218: 49–61. doi:[10.1007/s00221-012-3001-3](https://doi.org/10.1007/s00221-012-3001-3)

21.	Miyagishima S, Asaka T, Kamatsuka K, Kozuka N, Kobayashi M, Igarashi R, et al. Characteristics of antigravity spontaneous movements in preterm infants up to 3 months of corrected age. Infant Behav Dev. 2016;44: 227–239. doi:[10.1016/j.infbeh.2016.07.006](https://doi.org/10.1016/j.infbeh.2016.07.006)

22.	Marchi V, Belmonti V, Cecchi F, Coluccini M, Ghirri P, Grassi A, et al. Movement analysis in early infancy: Towards a motion biomarker of age. Early Hum Dev. 2020;142: 104942. doi:[10.1016/j.earlhumdev.2019.104942](https://doi.org/10.1016/j.earlhumdev.2019.104942)

23.	Kim J-W, Choi J-Y, Ha E-J, Choi J-H. Human pose estimation using MediaPipe pose and optimization method based on a humanoid model. Appl Sci. 2023;13: 2700. doi:[10.3390/app13042700](https://doi.org/10.3390/app13042700)

24.	Ben Gamra M, Akhloufi MA. A review of deep learning techniques for 2D and 3D human pose estimation. Image Vis Comput. 2021;114: 104282. doi: [10.1016/j.imavis.2021.104282](https://doi.org/10.1016/j.imavis.2021.104282)

25.	Stenum J, Cherry-Allen KM, Pyles CO, Reetzke RD, Vignos MF, Roemmich RT. Applications of pose estimation in human health and performance across the lifespan. Sensors (Basel). 2021;21: 7315. doi:[10.3390/s21217315](https://doi.org/10.3390/s21217315)

26.	Sermpon N, Gima H. A systematic review of the measurement of infant posture and movement using image or video data analysis | Archives of allied health sciences. Arch AHS. 2022;34: 1–15.

27.	Cao Z, Hidalgo G, Simon T, Wei S-E, Sheikh Y. OpenPose: Realtime multi-person 2D pose estimation using part affinity fields. IEEE Trans Pattern Anal Mach Intell. 2021;43: 172–186. doi:[10.1109/TPAMI.2019.2929257](https://doi.org/10.1109/tpami.2019.2929257)

28.	Hesse N, Sebastian Schroeder A, Müller-Felber W, Bodensteiner C, Arens M, G. Hofmann U. Markerless motion analysis for early detection of infantile movement disorders. In: Eskola H, Väisänen O, Viik J, Hyttinen J, editors. EMBEC & NBC 2017. Singapore: Springer; 2018. pp. 197–200. doi:10.1007/978-981-10-5122-7_50

29.	Reich S, Zhang D, Kulvicius T, Bölte S, Nielsen-Saines K, Pokorny FB, et al. Novel AI driven approach to classify infant motor functions. Sci Rep. 2021;11: 1–13. doi:[10.1038/s41598-021-89347-5](https://doi.org/10.1038/s41598-021-89347-5)

30.	Marcroft C, Khan A, Embleton ND, Trenell M, Plötz T. Movement recognition technology as a method of assessing spontaneous general movements in high risk infants. Front Neurol. 2015;5: 284. doi:[10.3389/fneur.2014.00284](https://doi.org/10.3389%2Ffneur.2014.00284)

31.	Pose landmark detection guide | MediaPipe. In: Google for Developers [Internet]. [cited 6 Sep 2023]. Available: https://developers.google.com/mediapipe/solutions/vision/pose_landmarker

2. *“The description of the methodology, particularly the video recording and data collection procedures, lacks comprehensive details. For instance, there is no mention of the camera specifications used for recording, which could significantly impact the pose estimation accuracy. Additionally, information on the calibration process for the 2D pose estimation tool and the handling of potential errors in pose estimation is missing.”*

**In light of your comment, we have revised the section “Participants and video collection procedure” in the methodology as follows:**

**Page 7, lines 156-173:**

Parents and guardians were instructed to record videos of the infants using the video function of their smartphones, setting the camera to a common configuration of 30 frames per s. First, they were asked to dress infants in a nappy or a bodysuit and place them on a firm mat in the supine position. Next, they had to hold the mobile camera and position it approximately 1 m above the infant, ensuring a top view covering all the infant's limbs. They were instructed to record a video for 2–3 min and avoid interacting with the infant while holding the mobile phone. No specific instructions were given to the guardians regarding when to start recording. Afterwards, the video file was securely transferred to the researcher through cloud storage using multiple security settings on Dropbox. Participants' autonomy and privacy were respected, with careful measures implemented to safeguard the confidentiality of infant data. Following the video collection phase, each video's duration was cut into 2-min clips of active infants lying supine, excluding crying or sleeping during the video. All videos were reformatted with a frame width and height of 1920 × 1080. Consequently, each video commenced by pressing the recording button. The initial 5 s of the video were cut off and subsequently maintained for 2 mins thereafter. Videos were excluded due to visibility issues, such as infants energetically moving their limbs beyond the camera frame, leading to the program's inability to detect corresponding landmark coordinates. Furthermore, if the program failed to execute the code in its entirety, resulting in an incomplete analysis, the video was excluded.

3. *“The sample size appears limited (20 infants), raising concerns about the generalizability of the findings. Also, the criteria for selecting the infants and the videos used for analysis are not adequately detailed.”*

**We asked for volunteers and recruited a total of 20 infants. The number of participants in this study is not sufficiently large; however, we believe it is significant as we assessed them longitudinally over a specific period of time (94 videos in total). The inclusion criteria for infant selection included term infants without any diseases or abnormalities. Videos were excluded due to visibility issues and data extraction failure.**

**Page 6, lines 141-149, and Page 7, lines 150-173:**

**Participants and video collection procedure**

This study collected video recordings of term infants aged from 8 to 16 weeks PTA without any diagnosed diseases or abnormalities. Participant recruitment was performed using two methods: direct contact with parents or guardians taking care of young infants and asking if they wished to participate in this research voluntarily via email or in person, or via poster invitations in parent support groups on social media asking for voluntary participation. At the onset, the study details were conveyed individually to each parent or guardian. Comprehensive information sheets were provided, and they were actively encouraged to seek clarification. Upon gaining a thorough understanding of the process, they proceeded to electronically complete a consent form, specifying the participant's name, their relationship with the infant, the signatures of the parents or guardians, and the date. Then, we asked parents or guardians to send a video of their infants through their mobile phones using an online private cloud storage that was secure. This study was approved by the relevant human research ethics committee (approval number: 21092). The recruitment period was from 24th March 2022 to 28th February 2023.

Parents and guardians were instructed to record videos of the infants using the video function of their smartphones, setting the camera to a common configuration of 30 frames per s. First, they were asked to dress infants in a nappy or a bodysuit and place them on a firm mat in the supine position. Next, they had to hold the mobile camera and position it approximately 1 m above the infant, ensuring a top view covering all the infant's limbs. They were instructed to record a video for 2–3 min and avoid interacting with the infant while holding the mobile phone. No specific instructions were given to the guardians regarding when to start recording. Afterwards, the video file was securely transferred to the researcher through cloud storage using multiple security settings on Dropbox. Participants' autonomy and privacy were respected, with careful measures implemented to safeguard the confidentiality of infant data. Following the video collection phase, each video's duration was cut into 2-min clips of active infants lying supine, excluding crying or sleeping during the video. All videos were reformatted with a frame width and height of 1920 × 1080. Consequently, each video commenced by pressing the recording button. The initial 5 s of the video were cut off and subsequently maintained for 2 mins thereafter. Videos were excluded due to visibility issues, such as infants energetically moving their limbs beyond the camera frame, leading to the program's inability to detect corresponding landmark coordinates. Furthermore, if the program failed to execute the code in its entirety, resulting in an incomplete analysis, the video was excluded.

4. *“The discussion section requires expansion. It should not only interpret the results but also compare them with existing literature, discussing how this study contributes to the current understanding of infant movement analysis.”*

**In accordance with your comment, we have revised the Discussion and added more details comparing our research to the literature.**

**Pages 15-18, lines 352-432:**

In this study, we collected videos of healthy infants aged between 8- and 16- weeks PTA (recording by parents or guardians) and observed the occurrence of MTM within 10 MTM items (Lower MTM included FF, FL, and pedipulation, whereas Upper MTM encompassed HH, HF, HM, HT, HL, manipulation, and fiddling). We then utilised 2D pose estimation to extract landmark coordinates, enabling the calculation of distances and areas. There were three major findings of this study: 1) in the upper MTM, 'HT' was the most frequently observed motor behaviour, whereas in the lower MTM, 'FF' was the most prevalent; 2) we identified weak correlations between the calculated distance and MTM occurrence values; and 3) significant differences were observed in both the whole area and lower part area between videos showing MTM and those without MTM within the Lower MTM category.

MTM was considered the initial antigravity movement behaviour that would eventually develop into goal-directed movements, i.e. reaching and grasping, feeding, and walking. In this study, the FF and HT movement patterns were most frequently observed for the lower and upper limbs, respectively. In typical infants, lower limb movement normally begins with leg lifting and then progresses to foot contact and exploration with various patterns of leg movements [33,34]. In this study, three out of ten MTM items exhibited the development of lower limb movements over time. Consequently, the frequency of FF occurrences was notably high in this age group. This observation is consistent with a previous study [14], which demonstrated a similar increase in the frequencies of FF by age. FF movement presented as an early antigravity movement; the absence of FF potentially identifies neurodevelopmental problems during early infancy. Ferrari et al. [35] studied the use of FF movements for supporting FMs by assessing both FMs and FF occurrence in infants with hypoxic-ischemic encephalopathy, describing the sensitivity and specificity for CP prediction individually and when pooling the two factors together. The result showed the sensitivity and specificity of both patterns to predict CP was 100% and 73%, respectively. This suggests that FF could serve as a supportive tool, emphasizing the need for further exploration of various FF movements. When considering FF subtypes, such as plant-to-plant contact, plant-to-tibial margin of the contralateral foot, plant-to-dorsal part of the contralateral foot, feet crossing, and pedipulation, more specific information about infant development can be obtained [35]. Meanwhile, the FL components exhibit higher development, involving lifting the leg and crossing over to touch the opposite side. The repetition of this movement pattern could lead to the rolling component [36]. HT is a fundamental motor behaviour observed in infants when they exhibit fewer uncontrolled movements (i.e. swipes and oscillations) or it could be from ATNP. Among the seven upper limb MTM items, it is challenging to deduce that HT is an antigravity movement of the entire arm; certain components involve lifting the forearm from the floor and placing it against the body. However, infants brought their arms and hands to contact their body to initiate self-exploration. This motor behaviour can enhance somatosensory input, skill development, and subsequent behaviours [37,38].

Considering the correlation between the distance calculated from upper arm landmarks and MTM, occurrence values revealed significant positive correlations with left wrist landmarks and negative correlations with right wrist landmarks. These associations may be attributed to the infant’s posture, where one hand contacts the body while the other remains apart. After birth, infants experience a different environment as they are generally used to being in a flexion position with an inability to move against gravity. When they are lying on their back, they may show a variety of motor and posture patterns [9]; head turn aside, arm sweeping, or leg kicking unintentionally. In this study, the different directions of upper limb landmarks may represent the asymmetry which is commonly found in early infant development. In addition, the presence of ATNP may influence this outcome, as ATNP occurs when the infant rotates their head to the side while extending one arm and leg and flexing the other arm and leg on the opposite side [14,39]. As infant postural control improves with age, various MTM items, such as HH, begin to emerge and develop symmetrical movement patterns [40].

In this study, we calculated area using wrist and ankle landmarks. According to the hypothesis, the presence of MTM may decrease these areas. In the lower-limb MTM group, significant differences were observed in the whole and lower area between videos that showed MTM and no MTM. The lower limb MTM category encompassed FF, FL, and pedipulation movements. These specific movements involve positioning the ankle and foot closer to each other, resulting in a smaller area compared with having the legs apart. Infants require strength, skill, and experience to bring their feet together while lifting their legs [41,42]. In the upper-limb MTM group, there were no significant difference in all areas between videos showing MTM and no MTM. However, when considering the upper part area, the group displaying MTM exhibits a smaller area compared with those not showing MTM. This category encompasses seven MTM items that can be divided into two groups: one hand and both hands contacting body parts. These movements led to varied positions, including both wrist landmarks being close together and one landmark apart from another; the latter configuration resulted in a larger area. Further investigations could involve grouping arm positions and comparing corresponding areas to gain a more comprehensive understanding of the subject matter.

Automated pose estimation has been employed in many studies assessing early infant movement development. For instance, Marchi et al [43] reported high accuracy using OpenPose skeletal videos compared with visual assessments for GMA. Turner et al [44] applied a combination of deep learning and 2D pose estimation to classify children’s dexterity and position when interacting with a toy. In this study, we employed the open-source Google MediaPipe 2D pose estimation to identify features related to MTM in the supine position. Analysing distance and area revealed certain associations between computed values and MTM observation. Regarding the antigravity characteristic of MTM movement, a more in-depth investigation is warranted to uncover additional insights. Further complexity analysis, such as the development of 2D landmarks into 3D pose estimation [23], may be necessary to enhance our understanding. However, assessing MTM movement in infants aged between 3 and 4 months could be practical in clinical settings [14]. The implementation of pose estimation may be useful for infants in remote areas or facing challenges with transportation to hospitals or rehabilitation centres.

(References)

9.	Ferrari F, Plessi C, Lucaccioni L, Bertoncelli N, Bedetti L, Ori L, et al. Motor and postural patterns concomitant with general movements are associated with cerebral palsy at term and fidgety age in preterm infants. J Clin Med. 2019;8: 1189. doi: [doi.org/10.3390/jcm8081189](https://doi.org/10.3390/jcm8081189)

14.	Lucaccioni L, Bertoncelli N, Comini M, Martignoni L, Coscia A, Lugli L, et al. The ontogeny of limbs movements towards midline in healthy infants born at term. Early Hum Dev. 2021;155: 105324. doi:[10.1016/j.earlhumdev.2021.105324](https://doi.org/10.1016/j.earlhumdev.2021.105324)

23.	Kim J-W, Choi J-Y, Ha E-J, Choi J-H. Human pose estimation using MediaPipe pose and optimization method based on a humanoid model. Appl Sci. 2023;13: 2700. doi:[10.3390/app13042700](https://doi.org/10.3390/app13042700)

33.	Piek JP, Carman R. Developmental profiles of spontaneous movements in infants. Early Hum Dev. 1994;39: 109–126. doi:[10.1016/0378-3782(94)90160-0](https://doi.org/10.1016/0378-3782(94)90160-0)

34.	Deng W, Vanderbilt DL, Smith BA. Differences in spontaneous leg movement patterns between infants with typical development and infants at risk for developmental delay: Cross-sectional observation prior to sitting onset. J Mot Learn Dev. 2018;6: 101–113. doi:[10.1123/jmld.2016-0056](https://doi.org/10.1123/jmld.2016-0056)

35.	Ferrari F, Bedetti L, Bertoncelli N, Roversi MF, Della Casa E, Guidotti I, et al. Foot-to-foot contact among initial goal-directed movements supports the prognostic value of fidgety movements in HIE-cooled infants. Front Pediatr. 2022;9. doi:[10.3389/fped.2021.731021](https://doi.org/10.3389/fped.2021.731021)

36.	Kobayashi Y, Watanabe H, Taga G. Movement patterns of limb coordination in infant rolling. Exp Brain Res. 2016;234: 3433–3445. doi:10.1007/s00221-016-4741-2

37.	Babik I, Galloway JC, Lobo MA. Infants born preterm demonstrate impaired exploration of their bodies and surfaces throughout the first 2 years of life. Phys Ther. 2017;97: 915–925. doi:[10.1093/ptj/pzx064](https://doi.org/10.1093/ptj/pzx064)

38.	Kanazawa H, Yamada Y, Tanaka K, Kawai M, Niwa F, Iwanaga K, et al. Open-ended movements structure sensorimotor information in early human development. Proc Natl Acad Sci U S A. 2023;120: e2209953120. doi:[10.1073/pnas.2209953120](https://doi.org/10.1073/pnas.2209953120)

39.	Hamer EG, Hadders-Algra M. Prognostic significance of neurological signs in high-risk infants – A systematic review. Dev Med Child Neurol. 2016;58: 53–60. doi:[10.1111/dmcn.13051](https://doi.org/10.1111/dmcn.13051)

40.	Rocha NACF, Tudella E. The influence of lying positions and postural control on hand–mouth and hand–hand behaviors in 0–4-month-old infants. Infant Behav Dev. 2008;31: 107–114. doi:[10.1016/j.infbeh.2007.07.004](https://doi.org/10.1016/j.infbeh.2007.07.004)

41.	Galloway JC, Thelen E. Feet first: Object exploration in young infants. Infant Behav Dev. 2004;27: 107–112. doi:[10.1016/j.infbeh.2003.06.001](https://doi.org/10.1016/j.infbeh.2003.06.001)

42.	Discovery of motor development: A tribute to Esther Thelen. [cited 19 Jun 2023]. Available: https://psycnet.apa.org/fulltext/2014-44019-007.html

43.	Marchi V, Hakala A, Knight A, D’Acunto F, Guzzetta A, Scattoni ML, et al. Automated pose estimation captures key aspects of general movements at eight to 17 weeks from conventional videos. Acta Paediatr. 2019;108: 1817–1824. doi:[10.1111/apa.14781](https://doi.org/10.1111/apa.14781)

44.	Turner A, Hayes S, Sharkey D. The classification of movement in infants for the autonomous monitoring of neurological development. Sensors. 2023;23: 4800. doi: [10.3390/s23104800](https://doi.org/10.3390/s23104800)

5. *“The study's limitations are not thoroughly discussed.”*

**In accordance with your comment, we have revised the study’s limitation as follows:**

**Page 18, lines 433-434, and Page 19, lines 435-451:**

This study had some limitations. First, video recording was done by a parent or guardian; hence, it was common to have some interferences that could not be avoided due to their presence when approaching infants of this age. Infants might react and need a response from the person who was taking a video. Some of them stared at the mobile phone and did not show any movement. Second, despite efforts to maintain a perpendicular position for handheld smartphones during recording, slight movements or changes in camera angles owing to the camera-holding hand were possible. However, since the features calculated in this study are angular information obtained by connecting markers with lines and distance information corrected by body length, we believe that their influence was minimized. Hand-holding a camera may have been a convenient and realistic method for parents or caregivers. However, if they had to set up a camera tripod or control the camera similar to a laboratory setting, this may have become burdensome. Improving the capacity to adapt to factors such as camera angle and occlusion is essential for optimizing the functionality of a lightweight, parent-controlled system in identifying neuromotor delays in infants. This enhanced ability ensures more robust performance, enabling accurate assessments even in challenging conditions. Third, the sample size appears to be limited, and as a result, the presented findings may not accurately represent all infants. Further study is required with a larger number of infants and an extended duration of follow-up to thoroughly assess the effectiveness of the 2D pose estimation method in extracting values related to MTM.

6. *“Some references are outdated or not directly relevant to the study's focus. Updating these with more recent and pertinent studies would strengthen the background and support the research.”*

**In accordance with your comment, we have reviewed and revised our references. The references are now listed as per the revised manuscript.**

**Reviewer #3**

*“The study seems an interesting novel approach which is appreciable, yet there are major concerns regarding the scientific soundness ,validity, reliability and external journalisability of the study.
The authors seem to be representing an over simplified and speculative version of the findings, which may lead to huge erreneous impressions to the scientific community.
There is no mention of the study method, settings, sample size estimation and validity of the tools used in the scientific methodology of the current study.
In fact the previous literature which have been referred to as the hypothesis generating resource,e.g ref 11 Ferrari et al, also have major limitations in terms of an appropriate size of sample used in their study to be used for anaysis of a statistically and clinically relevant study.Further the previous authors have clearly mentioned about the unreliability of the time line used for analysis in their study and have recommended strongly for the recordings at later time period of development as a future recommendation, which yet has been ignore in the current study method.
The population used in the previous literature are the children suffering from hypoxic ischemic encephalopathy on which the findings of investigation are based upon, the current study method has conveniently ignored a very important confounder that is the neurological health status of the infant in this investigation including that could have talked of a possibly entire different scenario.
In my humble opinion the study does not seen fit for publication in the current format.
In fact at any other point of time if the authors are interested to submit this to any other journal it must be referred to as preliminary observations only or the scientific soundness of the study methodology needs to be upgraded and then revised findings should be published at a later time.”*

**We have made significant revisions to enhance the clarity of the Introduction, Methods, and Discussion sections. In order to focus on typical infants in this study, we have removed references to the Ferrari et al. study from the Introduction. Nevertheless, we have maintained discussions on foot-to-foot contact regarding our observed results. Your suggestions have been invaluable and have greatly helped improve our work. We hope our revisions address your concerns and improve the clarity, reproducibility, and clinical relevance of our study.**

**We received a lot of advice on the English wording of our draft; hence, we have once again engaged the services of an English proofreading service.**

**Thank you for your consideration. I look forward to hearing from you.**

**Sincerely,**

**Hirotaka Gima**

**Department of Physical Therapy, Graduate School of Human Health Sciences,**

**Tokyo Metropolitan University,**

**7-2-10 Higashi-Ogu, Arakawa-ku, Tokyo 116-8551, Japan**

**Email: gima@tmu.ac.jp**

**Tel: +81-3-3819-7302**
